# Defining nonfatal opioid overdoses using administrative health data in the United States and Canada: A scoping review

**DOI:** 10.1016/j.pmedr.2026.103594

**Published:** 2026-07-31

**Authors:** Megan E. Marziali, Silke Hansen, Kevin Y. Xu, Esther Rowan, Michelle Doering, Erin M. Annunziato, Robert S. Hogg, Silvia S. Martins

**Affiliations:** a Department of Epidemiology, Columbia University Mailman School of Public Health, NY, New York, United States; b British Columbia Centre for Excellence in HIV/AIDS, Vancouver, British Columbia, Canada; c Division of Addiction Science, Prevention and Treatment, Department of Psychiatry, Washington University School of Medicine; Washington University School of Public Health, St Louis, MO, United States; d McMaster University, Hamilton, Ontario, Canada; e Bernard Becker Medical Library, Washington University School of Medicine, St Louis, MO, United States; f Faculty of Health Sciences, Simon Fraser University, Burnaby, British Columbia, Canada

**Keywords:** Drug overdose, Opiate overdose, Scoping review, Epidemiology, International classification of diseases, Medical records

## Abstract

**Objective:**

To explore definitions of nonfatal opioid overdoses with study populations based in the United States and Canada, to improve nonfatal overdose monitoring.

**Methods:**

We searched five databases: PubMed, Medline, Web of Science, Embase, and PsycInfo, including studies published between January 2013–October 2024. We combined overdose-related terms (e.g., overdose, poisoning) and hospital-related terms (e.g., diagnosis code, International Classification of Diseases [ICD], electronic health records) and extracted data from 214 studies that defined opioid overdoses.

**Results:**

Most studies reported on opioid overdoses due to any opioid (*n* = 175). Studies varied in terms of specific ICD codes. Among studies using ICD, Tenth Revision, Clinical Modification (ICD-10-CM) codes (*n* = 79), key sources of variation included intent specifications and encounter type; nearly one-third did not specify whether codes reflected an initial encounter, a subsequent visit, or sequelae (*n* = 24, 30.8%). Of studies that specified, most used all three encounter types (*n* = 27, 34.6%) or initial only (*n* = 21, 26.9%). Few specified both initial and sequelae (*n* = 4, 5.1%) or initial and subsequent encounters (n = 2, 2.6%). Similar patterns were observed across all opioid subtype definitions.

**Conclusions:**

We observed substantial variation in definitions. Detailed, standardized reporting methods when applying diagnostic codes may improve consistency and transparency and could improve public health monitoring.

## Highlights

• We found variation in diagnostic codes used to define nonfatal opioid overdoses.
• Studies varied inclusion of diagnostic codes by intent and encounter types.
• Detailed, standardized reporting methods are needed for improved monitoring.

## 1 Introduction

Drug overdoses, often involving opioids, remain a public health concern in the United States (US) and Canada. In the US, fatal drug overdoses exceeded 100,000 for the first time from 2020 to 2021 (Centers for Disease Control and Prevention (CDC), 2026a); while recent data suggest a decrease in overdose deaths, deaths remain high (Centers for Disease Control and Prevention, 2024; Kiang and Humphreys, 2025). Similarly, drug overdose mortality rates in Canada reached a high of 21.2 per 100,000 persons in 2023 (Government of Canada, 2026). Evaluating the extent of the drug overdose crisis solely through mortality excludes crucial context: nonfatal overdoses. Estimates suggest that nonfatal overdose rates in the US surpass those of fatal overdoses (Centers for Disease Control and Prevention (CDC), 2026b; Marziali et al., 2025). Factors driving recent declines in opioid overdose rates in the US, such as the widespread use of naloxone (Centers for Disease Control and Prevention (CDC), 2025a; Stringfellow et al., 2026), may not reduce the number of nonfatal opioid overdoses. As nonfatal opioid overdose is associated with increased risk of subsequent fatal overdose (Olfson et al., 2018; Hood et al., 2023), preventing nonfatal opioid overdoses is a public health priority.

Overdoses must be consistently measured to accurately surveil rates; however, measurement differs across data sources. For example, data collected through the Drug Overdose Surveillance and Epidemiology (DOSE) system can vary by jurisdiction; through DOSE-SYS, 47 states and the District of Columbia (DC) submit syndromic surveillance data (e.g., emergency department visits) (Centers for Disease Control and Prevention (CDC), 2026c). Through DOSE-DIS, a total of 34 states and DC submit discharge data from emergency department visits; of those, 31 states and DC submit emergency department and inpatient hospitalization discharges, while three submit inpatient data only (Centers for Disease Control and Prevention (CDC), 2026b). This variation in data sources between states can complicate efforts to identify regions in greater need of resources to prevent nonfatal overdoses. Additional guidance from other sources, such as the Council of State and Territorial Epidemiologists, proposes the inclusion of a wide array of sources, such as emergency department visits, emergency medical services, poison control centers, and criminal legal system data, to define nonfatal overdoses (Rodriguez et al., 2025; Council of State and Territorial Epidemiologists (CSTE), 2025a; Council of State and Territorial Epidemiologists (CSTE), 2025b). Definitions encompassing differing data add complexity when comparing the occurrence of nonfatal overdoses by introducing measurement heterogeneity across contexts, which may need to be considered when conducting public health monitoring of nonfatal overdose rates across geographic areas.

Definitions of nonfatal overdoses using electronic health records also vary. Electronic health records rely on the International Classification of Diseases (ICD) codebook, a globally standardized diagnostic tool developed by the World Health Organization to classify various health conditions (World Health Organization (WHO), 2025). The codebook evolved over time, with the 9th version (ICD-9) in use until approximately 2006 in Canada and 2015 in the US, when health systems transitioned to the ICD-10 (Canadian Institute for Health Information (CIHI), 2025a; Centers for Medicare and Medicaid Services (CMS), 2025). Some countries adapted the ICD to their healthcare systems; Canada added a Canadian modification (ICD-10-CA) (Canadian Institute for Health Information (CIHI), 2025b), and the US added a Clinical Modification (ICD-10-CM) (Centers for Disease Control and Prevention (CDC), 2025b). The ICD-10-CM allows for additional elements to be embedded into the ICD code. Most relevant to overdoses is the addition of a 6th or 7th digit to indicate intentionality (1–6), and a final letter to indicate encounter type (A, D, S) (Centers for Disease Control and Prevention (CDC), 2025b; Vivolo-Kantor et al., 2021). Intentionality is defined as unintentional (1), intentional (2), assault (3), undetermined (4), adverse effect (5), or underdosing (6). Encounter types include an initial encounter (A), subsequent encounter (D), or sequela (S) (Centers for Disease Control and Prevention (CDC), 2025b; Vivolo-Kantor et al., 2021). These additional data elements add to the base ICD code, indicating specific health conditions. For example, the code for accidental heroin poisoning at the initial encounter is T40.1X1A. Thus, ICD-10-CM codes can vary by: (1) the base ICD code defining the health condition, (2) intentionality, and (3) encounter types.

Though ICD codebooks provide detailed instructions and guidelines for use (Centers for Disease Control and Prevention (CDC), 2025b), assigning diagnostic codes involves subjectivity. Moreover, subjectivity is introduced when researchers include diagnostic codes to define opioid overdoses – particularly when considering the wide sources of variability introduced by the ICD-10-CM system (Vivolo-Kantor et al., 2021). Researcher discretion dictates interpretation of diagnostic codes and reporting. This can lead to variability in case definitions across studies, potentially influencing comparability of findings. Transitions between ICD versions can further compound this variability, as differences in diagnostic code structure, specificity, and classification rules may alter inclusion and exclusion criteria, thereby affecting study outcomes.

We aim to explore definitions of nonfatal opioid overdoses applied in research articles with US and Canadian study populations. Specifically, we focus our search on studies applying or developing definitions for nonfatal opioid overdoses using electronic health records or administrative health data, such that we can examine (1) the breadth of diagnostic codes used across studies, (2) specific aspects of ICD-10-CM codes, and (3) methodologies for including diagnostic codes (e.g., the number of case fields searched). We aim to provide recommendations for methodological reporting to support the field in moving towards a cohesive definition of nonfatal opioid overdoses and improve epidemiological monitoring.

## 2 Methods

We conducted a scoping review of published literature to examine definitions of nonfatal opioid overdoses using administrative health data. Clarifying definitions in the literature is a well-supported rationale for conducting a scoping review instead of a systematic review, thus supporting our decision to apply this methodology (Munn et al., 2018). We include studies published from January 2013–October 2024. We evaluated studies from 2013 onwards to align with the most recent wave of opioid overdose deaths in the US, attributable to synthetic opioids (Centers for Disease Control and Prevention, 2025). We present definitions categorized by opioid overdose type: (1) any opioids, (2) heroin, (3) prescription opioids, (4) other categorizations of opioids (including synthetic opioids, such as fentanyl), and (5) non-heroin opioids. This scoping review followed the Preferred Reporting Items for Systematic Reviews and Meta-Analyses Extension for Scoping Reviews Guidelines (Tricco et al., 2018) and guidance for conducting scoping reviews (Peters et al., 2015; Peters et al., 2020). This review protocol was published on Open Science Framework (Marziali et al., 2024).

We conducted the search in October 2024 across five databases selected for comprehensiveness and relevance to the research question: PubMed, Medline, Web of Science, Embase, and PsycInfo. We combined keywords and subject headings for overdose-related terms (e.g., overdose) with hospital-related terms (e.g., diagnosis code, International Classification of Disease, electronic health records, data collection). An English-language filter, a date-limit of 2013-current, and a publication-type limit to exclude case reports were applied. The full search strategy for all databases is available in **Supplemental Table 1**.

Studies were eligible for inclusion if they: (1) explicitly measured nonfatal opioid overdose, either through operationalizing nonfatal opioid overdose as the exposure or outcome, or cohort characteristic, including syndromic surveillance data, (2) were published in English, (3) included data from the US or Canada, and (4) were original, empirical research.

Studies were excluded if they: (1) were qualitative research, case reports, literature reviews, conference abstracts or empirical research which measured nonfatal overdoses via self-report or survey data, (2) did not define nonfatal opioid overdoses, (3) were animal studies, (4) used data exclusively from poison control centers, 911 calls, or emergency medical services not linked with hospital data. These latter sources capture suspected overdoses, and without clinical confirmation, we cannot determine whether these events constituted overdoses. We also excluded (5) overdoses/poisonings not attributable to opioids, such as non-specific definitions (e.g., “any drug”), as the degree of variation in these definitions merits a separate review. We excluded (7) studies using terminology such as “drug-related hospitalizations”, or otherwise not explicitly describing overdose events as an overdose or drug poisoning. Similar to our rationale for excluding data from poison control centers, we were cognizant of introducing artificial measurement heterogeneity by including studies referring to differing, though potentially similar, constructs. We excluded (6) opioid overdoses/poisonings among pediatric populations, which included studies where the authors identified their population as pediatric, or the study population was restricted to adolescents. Lastly, we excluded (7) opioid poisonings/overdoses exclusively described as suicide attempts, meaning the researchers used the terminology “suicide attempt” rather than “nonfatal opioid overdose”. This was done to avoid introducing artificial variation in definitions due to differing constructs.

Overall, 16,490 articles were imported into Covidence (Veritas Health Innovation, 2026); 9524 were duplicates. Of the resulting 6966 articles, reviewers conducted the title and abstract screening independently, followed by full-text screening. Articles were excluded during title/abstract screening when they did not meet the inclusion criteria and were retained if they seemed to meet criteria or if it was unclear whether they met criteria; reviewers erred on inclusion. To be included in the study, two reviewers must have voted affirmatively (MEM, SH, ER, EMA). Conflicts were resolved by a third reviewer or by the reviewers meeting to discuss and resolve any discrepancies. Once full-text screening was finalized, reviewers extracted relevant data. Data charting was completed through Covidence; an initial data charting template was developed to determine which variables were appropriate for extraction. We piloted the data extraction template with a subset of papers and met to discuss the extraction process and ensure consistency across reviewers. See **Supplemental Table 2** for data charting elements. In brief, we extracted information relevant to characteristics of included studies (e.g., study period, country) and how nonfatal overdoses were defined and measured (e.g., data used to define opioid overdoses, ICD codes used, categories of opioids, case fields used). Data charting was completed independently; once data were imported into Excel, two authors reviewed all extracted data and double-checked elements against information reported in the source manuscripts to minimize possible human error introduced by extracting ICD codes. Data were grouped by type of opioid into five categories identified through language that the authors used to describe opioid overdoses: any opioids, heroin, prescription opioids, other categorizations of opioids (including synthetic opioids, such as fentanyl), and non-heroin opioids. Once the extracted data was finalized in Excel, we used R to summarize descriptive statistics.

## 3 Results

**Fig. 1:**
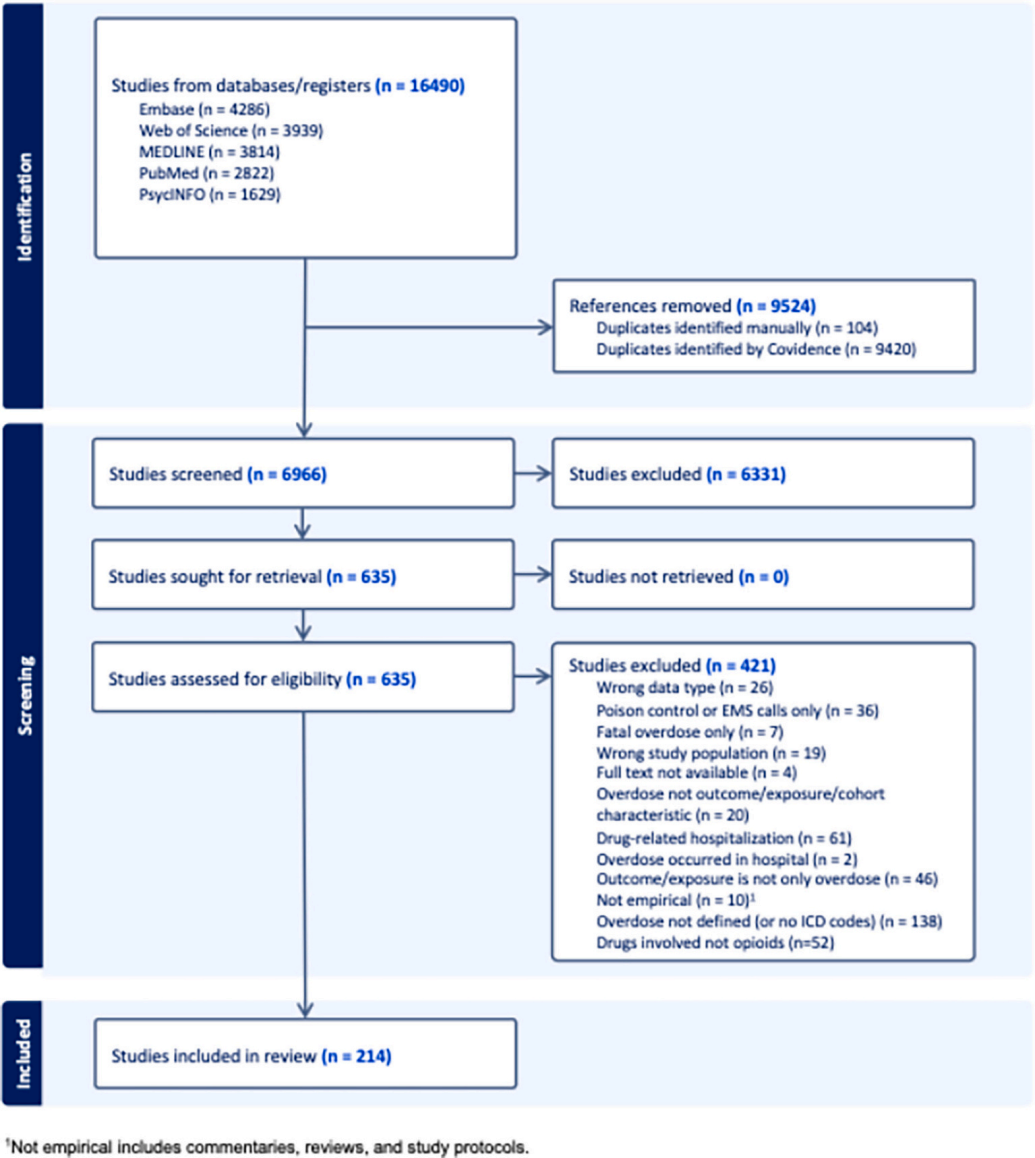
Diagram depicting the literature review process and reasons for study exclusions to explain all studies included in the current review, which included studies published between 2013 and 2024 in the United States and Canada.

We excluded 6331 articles at the title/abstract screening stage and 421 articles at the full-text screening phase. Reasons for exclusion are detailed in Fig. 1. We extracted data from 214 studies. While we originally intended to examine windows of inclusion for which diagnostic codes are defined as a single overdose event (e.g., two ICD codes related to drug poisoning in one day), most studies did not report this metric; thus, we do not examine this measure further. All extracted data are available upon request.

Of the 214 extracted studies, the most common subtype involved any opioids (*n* = 175); 33 studies defined heroin overdoses, 19 defined prescription opioid overdoses, 16 defined non-heroin overdoses, and seven studies defined overdoses occurring via another opioid subtype or combination (e.g., synthetic opioids, etc.). These categories were not mutually exclusive (some studies applied multiple definitions) and were constructed using language from each study, such as: “heroin overdose,” “prescription opioid overdose,” and “non-heroin overdose.” Most studies included data from the US (*n* = 199), with few studies originating from Canada (*n* = 15).

### 3.1 Opioid overdose definitions

We provide a detailed account of the ICD codes used to define opioid overdoses below. Where validation studies used multiple definitions, we report either the recommended definition or the simplest definition provided. While some studies described using ICD-10 codes, they reported codes consistent with ICD-10-CM formatting – we interpret these as ICD-10-CM codes.

*Any opioid overdoses*. Of all studies identified, 175 defined opioid overdoses. Most studies (*n* = 67) defined opioid overdoses using ICD-9 codes, followed by ICD-10-CM (*n* = 79), ICD-9-CM (*n* = 57), and ICD-10 (*n* = 39). Few studies used ICD-10-CA codes (*n* = 4). Note that, while many studies described reporting ICD-10 codes (*n* = 66), 27 listed diagnostic codes consistent with the formatting for ICD-10-CM; we are interpreting these as ICD-10-CM codes. The prevalence of common diagnostic codes and their specific definitions are presented in Table 1. Diagnostic codes appearing in fewer than 10 studies are not presented in Table 1 but are listed in **Supplemental Table 3**. A common definition among studies that only applied ICD-10 codes involves defining any opioid overdose via T40.0, T40.1, T40.2, T40.3, T40.4, and T40.6; similarly, among studies applying ICD-9 or ICD-9-CM codes, many definitions involved the codes 965.00, 965.01, 965.02, 965.09, E850.0, E850.1, and E850.2 (**Supplemental Table 3**). While uncommon, some studies included codes for opioid-related disorders, such as F11.1 (opioid abuse), F11.2 (opioid dependence), and F11.9 (opioid use) (**Supplemental Table 3**).

**Table 1:** ICD codes used to define any opioid overdose (*n* = 175), with the 10 most frequently used codes presented for interpretability, from studies identified in this review that included studies published between 2013 and 2024 in the United States and Canada.

ICD version	ICD code	Definition	Prevalence
ICD-9	E850.1	Accidental poisoning by methadone	88%
	E850.2	Accidental poisoning by other opiates and related narcotics	84%
	E850.0	Accidental poisoning by heroin	79%
	965.09	Poisoning by other opiates and related narcotics	78%
	965.02	Poisoning by other opiates and related narcotics	76%
	965.00	Poisoning by opium, unspecified	76%
	965.01	Poisoning by heroin	72%
	965.0	Poisoning by opiates and related narcotics	33%
	E935.0	Heroin causing adverse effects in therapeutic use	21%
	E935.2	Other opiates and related narcotics causing adverse effects in therapeutic use	21%
ICD-9-CM	965.02	Poisoning by other opiates and related narcotics	95%
	965.09	Poisoning by other opiates and related narcotics	93%
	965.00	Poisoning by opium, unspecified	91%
	E850.1	Accidental poisoning by methadone	91%
	E850.2	Accidental poisoning by other opiates and related narcotics	91%
	965.01	Poisoning by heroin	82%
	E850.0	Accidental poisoning by heroin	80%
	965.0	Poisoning by opiates and related narcotics	21%
	E935.1	Methadone causing adverse effects in therapeutic use	14%
	E935.2	Other opiates and related narcotics causing adverse effects in therapeutic use	13%
ICD-10	T40.2	Poisoning by, adverse effect of and underdosing of other opioids	87%
	T40.0	Poisoning by, adverse effect of and underdosing of opium	85%
	T40.1	Poisoning by and adverse effect of heroin	85%
	T40.3	Poisoning by, adverse effect of and underdosing of methadone	85%
	T40.4	Poisoning by, adverse effect of and underdosing of other synthetic narcotics	85%
	T40.6	Poisoning by, adverse effect of and underdosing of other and unspecified narcotics	67%
	F11.1	Mental and behavioral disorders due to use of opioids: harmful use	8%
	F11.2	Mental and behavioral disorders due to use of opioids: dependence syndrome	8%
	F11.3	Mental and behavioral disorders due to use of opioids: withdrawal state	5%
	F11.4	Mental and behavioral disorders due to use of opioids: withdrawal state with delirium	5%
ICD-10-CM	T40.0 × 1	Poisoning by opium, accidental (unintentional)	82%
	T40.2 × 1	Poisoning by other opioids, accidental (unintentional)	80%
	T40.3 × 1	Poisoning by methadone, accidental (unintentional)	80%
	T40.0 × 4	Poisoning by opium, undetermined	79%
	T40.2 × 4	Poisoning by other opioids, undetermined	77%
	T40.3 × 4	Poisoning by methadone, undetermined	73%
	T40.4 × 1	Poisoning by, adverse effect of and underdosing of other synthetic narcotics (unintentional)	73%
	T40.1 × 1	Poisoning by heroin, accidental (unintentional)	70%
	T40.4 × 4	Poisoning by, adverse effect of and underdosing of other synthetic narcotics, undetermined	70%
	T40.0 × 2	Poisoning by opium, intentional self-harm	68%

ICD: International Classification of Diseases; ICD-9-CM: International Classification of Diseases, Ninth Edition, Clinical Modification; ICD-10-CM: International Classification of Diseases, Tenth Edition, Clinical Modification.

Note: ICD code definitions from icd9data.com and icd10data.com

Of the 79 studies reporting ICD-10-CM codes, the highest proportion (*n* = 27, 34.2%) included initial, subsequent, and encounters due to sequelae; several did not specify encounter types (*n* = 25, 31.6%), or specified initial encounters only (*n* = 21, 26.6%). Few studies specified initial and sequelae (*n* = 4, 5.1%), or initial and subsequent (n = 2, 2.5%). Of the 65 studies that reported diagnostic codes consistent with intent specifications, all included unintentional or undetermined intent specifications. Many (80%, *n* = 52) included intentional, and 73.8% (*n* = 48) included assault. Few studies included adverse effects (*n* = 14, 21.5%), and studies rarely included underdosing (*n* = 3, 4.6%).

Most studies (*n* = 129, 73.7%) did not describe whether they searched specific case fields for diagnoses. Of those that did, the most common schema was searching across all case fields (*n* = 30), followed by primary diagnoses only (*n* = 9). Seven studies searched a specified number of case fields (e.g., primary or secondary diagnoses).

*Heroin overdoses*. Most studies (*n* = 24) relied on ICD-9-CM codes to define heroin overdoses, followed by ICD-10-CM codes (*n* = 15), ICD-9 codes (*n* = 5), and ICD-10 codes (n = 2) (Table 2). There was little variation in the diagnostic codes used to define heroin overdoses; all studies reporting ICD-9 or ICD-9-CM codes used 965.01, defined as drug poisoning due to heroin (Table 3). A common case definition involved defining heroin overdoses using the codes 965.01 and E850.0 (Table 2). Most studies using ICD-10 or ICD-10-CM codes reported T40.1×[1–4], similarly used to define poisonings due to heroin (Table 3).

**Table 2:** Summary of studies that defined a heroin overdose (*n* = 33) identified in this review which included studies published between 2013 and 2024 in the United States and Canada.

Author	Year	Country	Study period	Codes to define a heroin overdose	Combine fatal and nonfatal?	Case fields used (e.g., primary, secondary)	ICD-10-CM only: encounter type
***ICD-9***							
Unick^S29^	2013	United States	1993–2009	965.01, E850.0	No mention of fatal overdoses	Primary only	
Unick^S28^	2014	United States	1992–2008	965.01, E850.0	No mention of fatal overdoses	Primary only	
Unick^S22^	2017	United States	2000–2014	965.01, E850.0	No mention of fatal overdoses	Any code field	
***ICD-9-CM***							
Alinsky^S19^	2020	United States	2009–2015	965.01, E850.0	No mention of fatal overdoses	Did not mention	
Castillo-Carniglia^S32^	2019	United States	2001–2014	965.01, E850.0	No mention of fatal overdoses	Other set number (e.g., first 5 positions)	
Guy^S27^	2018	United States	2010–2014	965.01, E850.0	No mention of fatal overdoses	Primary only	
Guy^S20^	2020	United States	2008–2015	965.01, E850.0	No mention of fatal overdoses	Did not mention	
Hsu^S31^	2017	United States	2001–2012	965.01, E850.0	No mention of fatal overdoses	Other set number (e.g., first 5 positions)	
Ji^S25^	2021	United States	2010–2014	965.01, E850.0	No mention of fatal overdoses	Any code field	
Mair^S33^	2018	United States	2004–2014	965.01, E850.0	No mention of fatal overdoses	Other set number (e.g., first 5 positions)	
Meiman^S26^	2015	United States	2003–2012	965.01, E850.0	No	Primary only	
Mosher^S21^	2017	United States	2007–2014	965.01, E850.0	No mention of fatal overdoses	Any code field	
Pear^S23^	2019	United States	2002–2014	965.01, E850.0	No mention of fatal overdoses	Any code field	
Peters^S17^	2018	United States	1998–2013	965.01, E850.0	No mention of fatal overdoses	Did not mention	
Sakhuja^S18^	2017	United States	2000–2013	965.01	No mention of fatal overdoses	Did not mention	
Sears^S30^	2019	United States	2001–2014	965.01	No mention of fatal overdoses	Primary only	
***ICD-10***							
El Ibrahimi^S16^	2023	United States	2018	T40.1	No; measured fatal ODs separately	No mention	
***ICD-10-CM***							
Krishnaswami ^S8^	2022	United States	2016–2017	T40.1 × 1, T40.1 × 2, T40.1 × 3, T40.1 × 4	No	Any code field	A
Peterson^a^^S13^	2019	United States	2016	T40.0 × 1, T40.0 × 2, T40.0 × 3, T40.0 × 4	No mention of fatal overdoses	Other set number (e.g., first 5 positions)	No specification
Slavova^S15^	2020	United States	Oct 2015 - Dec 2016	T40.1 × 1, T40.1 × 2, T40.1 × 3, T40.1 × 4	No mention of fatal overdoses	Any code field	A; D
***ICD-9, ICD-10***							
Hartung^S24^	2020	United States	2015–2017	965.01, E850.0; T40.1	Yes	Any code field	
***ICD-9-CM, ICD-10-CM***							
Brown^b^^S1^	2017	United States	2010–2016	965.01, E850.00; T40.1 × 1, T40.1 × 2, T40.1 × 3, T40.1 × 4	No mention of fatal overdoses	No mention	A
Casillas^S12^	2024	United States	2010–2020	965.01, E850.0; T40.1 × 1, T40.1 × 2, T40.1 × 3, T40.1 × 4	No	Did not mention	A; S; D
Chang^S11^	2019	United States	2015	965.01, E850.0; T40.1 × 1, T40.1 × 4	No mention of fatal overdoses	Other set number (e.g., first 5 positions)	A; S; D
Chen^S10^	2021	United States	2010–2018	965.01, E850.0; T40.1 × 1, T40.1 × 2, T40.1 × 3, T40.1 × 4	No mention of fatal overdoses	Any code field	A; S; D
Coffey^S4^	2020	United States	2012–2016	T40.1 × 1, T40.1 × 4	No mention of fatal overdoses	Other set number (e.g., first 5 positions)	A
Hartung^S9^^b^, ^c^	2022	United States	Jul 2014 - Dec 2017	965.01, E850.0; T40.1×,	No mention of fatal overdoses	No mention	A; S; D
Jairam^S14^	2020	United States	2006–2015	965.01; T40.1	No mention of fatal overdoses	Primary only	No specification
Krishnaswami^S3^	2020	United States	2013–2016	965.01, E850.0; T40.1 × 1, T40.1 × 2, T40.1 × 3, T40.1 × 4	No	Other: Primary for ICD-9 codes and any code field for ICD-10 codes	A
Lagisetty^S5^	2020	United States	Jan 2009 - Jun 2017	965.01, E850.0; T40.1 × 1, T40.1 × 4	No mention of fatal overdoses	Did not mention	A
Mayberry^S6^	2021	United States	2012–2016	965.01, E850.0; T40.1 × 1, T40.1 × 2, T40.1 × 3, T40.1 × 4	No mention of fatal overdoses	Any code field	A
Scholl^S7^	2021	United States	NA	965.01, E850.0; T40.1 × 1, T40.1 × 4	No mention of fatal overdoses	Did not mention	A
Vivolo-Kantor^S2^	2019	United States	2017–2018	965.01, E850.0; T40.1 × 1, T40.1 × 4	No mention of fatal overdoses	Did not mention	A

ICD: International Classification of Diseases; ICD-9-CM: International Classification of Diseases, Ninth Edition, Clinical Modification; ICD-10-CM: International Classification of Diseases, Tenth Edition, Clinical Modification.

a The definition for heroin overdose differs from the description of “poisoning by heroin” in the “opioid overdose: any type.” The authors may have meant to instead include T40.1 × 1–4.

b We are interpreting these ICD-10 codes as ICD-10-CM.

c We are interpreting T40.1× to mean all intent specifications (i.e., A, S, D).

**Table 3:** Prevalence of ICD codes reported by studies defining nonfatal heroin overdoses (n = 33) identified in this review, which included studies published between 2013 and 2024 in the United States and Canada.

**ICD version**	**ICD code**	**Definition**	**Prevalence**
ICD-9	965.01	Poisoning by heroin	100%
	E850.0	Accidental poisoning by heroin	100%^a^
ICD-9-CM	965.01	Poisoning by heroin	100%
	E850.0	Accidental poisoning by heroin	87%
ICD-10	T40.1	Poisoning by and adverse effect of heroin	100%
ICD-10-CM	T40.1 × 1	Poisoning by heroin, accidental (unintentional)	80%
	T40.1 × 4	Poisoning by heroin, undetermined	80%
	T40.1 × 2	Poisoning by heroin, intentional self-harm	47%
	T40.1 × 3	Poisoning by heroin, assault	47%
	T40.1	Poisoning by and adverse effect of heroin	13%
	T40.0 × 1	Poisoning by opium, accidental (unintentional)	7%
	T40.0 × 2	Poisoning by opium, intentional self-harm	7%
	T40.0 × 3	Poisoning by opium, assault	7%
	T40.0 × 4	Poisoning by opium, undetermined	7%

ICD: International Classification of Diseases; ICD-9-CM: International Classification of Diseases, Ninth Edition, Clinical Modification; ICD-10-CM: International Classification of Diseases, Tenth Edition, Clinical Modification.

Note: ICD code definitions from icd9data.com and icd10data.com

a One study reported E850.00.

Among the 15 studies that reported ICD-10-CM codes, a small majority (53.3%, *n* = 8)^S1–8^ used only initial encounters; few studies (26.7%, *n* = 4)^S9–12^ reported either initial, subsequent, or encounters due to sequelae. Only two studies^S13,14^ did not specify encounter types, and one study^S15^ used initial and subsequent encounters. Overall, 13 studies clearly specified intent within diagnostic codes: all 13 studies used unintentional or undetermined intent specifications, and eight studies also included intentional or assault specifications. One study did not list intent specifications,^S14^ and one study used notation suggesting that they included all intent specifications (e.g., T40.1×).^S9^

Studies varied in terms of whether they specified case fields for diagnoses; 13 studies did not mention case fields,^S1,2,5-7,9,12,13,16–20^ eight reported any diagnostic code field,^S8,10,15,21–25^ six used primary diagnostic fields,^S14,26–30^ and two specified either primary or secondary case fields.^S31,32^ One study specified different approaches for ICD-9 and ICD-10 codes,^S3^ and three used another set number of case fields or described using various case fields.^S4,11,33^

*Prescription opioid overdoses*. Overall, 19 studies defined prescription opioid overdoses; the majority (*n* = 15) of studies used ICD-9-CM codes,^S14,18,20,21,23,25,26,30-32,34–38^ while only three studies used ICD-10-CM codes (Table 4**)**.^S1,14,39^ All studies using ICD-9 or ICD-9-CM codes defined prescription opioid overdoses using 965.09 (Table 5). Commonly reported were 965.02 (methadone), 965.00 (opium), E850.2 (other opiates), and E850.1 (methadone) (Table 5), with a common definition comprising the codes 965.00, 965.02, 965.09, E850.1, and E850.2 (Table 4). ICD-10 and ICD-10-CM codes were less frequently used overall; of those that did use these diagnostic codes, most defined prescription opioid overdoses with T40.0×[1–4], T40.2×[1–4], and T40.3×[1–4], which refer to poisonings on account of opium, other opioids, and methadone, respectively (Table 5).

**Table 4:** Summary of studies that defined a prescription opioid overdose (*n* = 19) identified in this review, which included studies published between 2013 and 2024 in the United States and Canada.

Author	Year	Country	Study period	Codes to define a prescription opioid overdose	Combine fatal and nonfatal?	Case fields used (e.g., primary, secondary)	ICD-10-CM only: encounter type
***ICD-9***							
Unick^S29^	2013	United States	1993–2009	965.00, 965.02, 965.09, E850.1, E850.2	No mention of fatal overdoses	Primary only	
Unick^S22^	2017	United States	2000–2014	965.00, 965.02, 965.09, E850.1, E850.2	No mention of fatal overdoses	Any code field	
***ICD-9-CM***							
Burton^S37^	2019	United States	2010–2014	965.00, 965.02, 965.09, E850.1, E850.2	No mention of fatal overdoses	Did not mention	
Burton^S38^	2019	United States	2010–2014	965.00, 965.02, 965.09, E850.1, E850.2	No mention of fatal overdoses	Did not mention	
Castillo-Carniglia^S32^	2019	United States	2001–2014	965.00, 965.02, 965.09, E850.1, E850.2	No mention of fatal overdoses	Other set number (e.g., first 5 positions)	
Guy^S20^	2020	United States	2008–2015	965.00, 965.02, 965.09, E850.1, E850.2	No mention of fatal overdoses	Did not mention	
Hsu^S31^	2017	United States	2001–2012	965.00, 965.02, 965.09, E850.01, E850.2	No mention of fatal overdoses	Other set number (e.g., first 5 positions)	
Ji^S25^	2021	United States	2010–2014	965.00, 965.02, 965.09, E850.1, E850.2	No mention of fatal overdoses	Any code field	
Meiman^S26^	2015	United States	2003–2012	965.00, 965.02, 965.09, E850.1, E850.2	No	Primary only	
Modarai^S35^	2013	United States	2008–2010	965.02, 965.09, E850.1, E850.2	No	Did not mention	
Mosher^S21^	2017	United States	2007–2014	965.00, 965.09, E850.2	No mention of fatal overdoses	Any code field	
Pauly^S34^	2018	United States	2004–2014	965.00, 965.02, 965.09, E85.01, E85.02	No mention of fatal overdoses	Any code field	
Pear^S23^	2019	United States	2002–2014	965.00, 965.02, 965.09, E850.1, E850.2	No mention of fatal overdoses	Any code field	
Sakhuja^S18^	2017	United States	2000–2013	965.00, 965.02, 965.09	No mention of fatal overdoses	Did not mention	
Sears^S30^	2019	United States	2001–2014	965.00, 965.02, 965.09	No mention of fatal overdoses	Primary only	
Tadros^a^^S36^	2015	United States	2006–2011	965.02, 965.09, E850, E851, E852, E853, E854, E855, E856, E857, E858	No mention of fatal overdoses	Primary only	
***ICD-9, ICD-10-CM***							
Brown^S1^	2017	United States	2010–2016	965.00, 965.02, 965.09, E850.1, E850, T40.0 × 1, T40.0 × 2, T40.0 × 3, T40.0 × 4, T40.3 × 1, T40.3 × 2, T40.3 × 3, T40.3 × 4, T40.2 × 1, T40.2 × 2, T40.2 × 3, T40.2 × 4	No mention of fatal overdoses	Did not mention	A
Nguyen^S39^	2020	United States	2006–2018	965.0, 965.00, 965.02, 965.09, E850.1, E850.2, T40.0 × 1, T40.0 × 2, T40.0 × 3, T40.0 × 4, T40.2 × 1, T40.2 × 2, T40.2 × 3, T40.2 × 4, T40.3 × 1, T40.3 × 2, T40.3 × 3, T40.3 × 4, T40.4 × 1, T40.4 × 2, T40.4 × 3, T40.4 × 4	Yes	Did not mention	No specification
***ICD-9-CM, ICD-10-CM***							
Jairam^S14^	2020	United States	2006–2015	965.00, 965.02, 965.09, T40.0, T40.2, T40.3, T40.4, T40.6	No mention of fatal overdoses	Primary only	No specification

ICD: International Classification of Diseases; ICD-9-CM: International Classification of Diseases, Ninth Edition, Clinical Modification; ICD-10-CM: International Classification of Diseases, Tenth Edition, Clinical Modification.

a E codes used in conjunction with ICD codes to determine intent.

**Table 5:** Prevalence of ICD codes used to define nonfatal prescription opioid overdoses (n = 19) identified in this review, which included studies published between 2013 and 2024 in the United States and Canada.

ICD version	ICD code	Definition	Prevalence
ICD-9	965.00	Poisoning by opium, unspecified	100%
	965.02	Poisoning by methadone	100%
	965.09	Poisoning by other opiates and related narcotics	100%
	E850.1	Accidental poisoning by methadone	100%^a^
	E850.2	Accidental poisoning by other opiates and related narcotics	75%
	965.0	Poisoning by opiates and related narcotics	25%
	E850	Accidental poisoning by analgesics antipyretics and antirheumatics	25%
ICD-9-CM	965.09	Poisoning by other opiates and related narcotics	100%
	965.02	Poisoning by other opiates and related narcotics	93%
	965.00	Poisoning by opium, unspecified	87%
	E850.2	Accidental poisoning by other opiates and related narcotics	73%
	E850.1	Accidental poisoning by methadone	67%
	E850	Accidental poisoning by analgesics antipyretics and antirheumatics	7%
	E851	Accidental poisoning by barbiturates	7%
	E852	Accidental poisoning by other sedatives and hypnotics	7%
	E853	Accidental poisoning by tranquilizers	7%
	E854	Accidental poisoning by other psychotropic agents	7%
	E855	Accidental poisoning by other drugs acting on central and autonomic nervous system	7%
	E856	Accidental poisoning by antibiotics	7%
	E857	Accidental poisoning by other anti-infectives	7%
	E858	Accidental poisoning by other drugs	7%
ICD-10-CM	T40.0 × 1	Poisoning by opium, accidental (unintentional)	67%
	T40.0 × 2	Poisoning by opium, intentional self-harm	67%
	T40.0 × 3	Poisoning by opium, assault	67%
	T40.0 × 4	Poisoning by opium, undetermined	67%
	T40.2 × 1	Poisoning by other opioids, accidental (unintentional)	67%
	T40.2 × 2	Poisoning by other opioids, intentional self-harm	67%
	T40.2 × 3	Poisoning by other opioids, assault	67%
	T40.2 × 4	Poisoning by other opioids, undetermined	67%
	T30.3 × 1	Poisoning by methadone, accidental (unintentional)	67%
	T30.3 × 2	Poisoning by methadone, intentional self-harm	67%
	T30.3 × 3	Poisoning by methadone, assault	67%
	T30.3 × 4	Poisoning by methadone, undetermined	67%
	T40.4 × 1	Poisoning by, adverse effect of and underdosing of other synthetic narcotics (unintentional)	33%
	T40.4 × 2	Poisoning by, adverse effect of and underdosing of other synthetic narcotics, intentional self-harm	33%
	T40.4 × 3	Poisoning by, adverse effect of and underdosing of other synthetic narcotics, assault	33%
	T40.4 × 4	Poisoning by, adverse effect of and underdosing of other synthetic narcotics, undetermined	33%
	T40.0	Poisoning by, adverse effect of and underdosing of opium	33%
	T40.2	Poisoning by, adverse effect of and underdosing of other opioids	33%
	T40.3	Poisoning by, adverse effect of and underdosing of methadone	33%
	T40.4	Poisoning by, adverse effect of and underdosing of other synthetic narcotics	33%

ICD: International Classification of Diseases; ICD-9-CM: International Classification of Diseases, Ninth Edition, Clinical Modification; ICD-10-CM: International Classification of Diseases, Tenth Edition, Clinical Modification.

Note: ICD code definitions from icd9data.com and icd10data.com

a One study used the code E850.01, which we assume is meant to be E850.1.

Of the three studies using ICD-10-CM codes, two did not specify whether they included initial, subsequent, or sequelae encounters^S14,39^, and one study only included initial encounters.^S1^ Two studies included intent specifications; both included unintentional, undetermined, intentional, and assault specifications.^S1,39^

Studies were mixed concerning the specification of case fields used. Seven studies did not mention case fields,^S1,18,20,35,37–39^ five used any diagnostic code field,^S21–23,25,34^ five specified primary case fields only,^S14,26,29,30,36^ and two used either primary or secondary case fields.^S31,32^

*Overdoses involving other opioid subclasses*. Seven studies^S12,37,38,40–43^ defined unique subtypes of opioid overdoses (Table 6). This included defining methadone and other opioid overdoses,^S40–42^ illicit opioid overdoses,^S37,38^ and synthetic opioid overdoses (including fentanyl).^S12,43^ There were not a sufficient number of studies for each type of overdose to carefully examine variations in definitions. Only one study specifically defined fentanyl overdoses, using the ICD-10-CM code T40.41.^S43^

**Table 6:** Summary of studies that defined other subtypes of opioid overdose (n = 7) identified in this review, which included studies published between 2013 and 2024 in the United States and Canada.^a^

Author	Year	Country	Study period	Codes to define other types of opioid overdoses	Combine fatal and nonfatal?	Case fields used (e.g., primary, secondary)	ICD-10-CM only: encounter type
***ICD-9-CM***							
Burton^a^^S38^	2019	United States	2010–2014	965.01, E850.0	No mention of fatal overdoses	Did not mention	
Burton^a^^S37^	2019	United States	2010–2014	965.01, E850.0	No mention of fatal overdoses	Did not mention	
Fulton-Kehoe^b^^S40^	2015	United States	Apr 2006 - Dec 2010	965.09, 965.00, 965.02	No mention of fatal overdoses	Any code field	
***ICD-10-CM***							
Casillas^b^^S43^	2022	United States	Oct 2019 - Sep 2021	T40.4 × 1, T40.4 × 2, T40.4 × 3, T40.4 × 4, T40.4 × 5, T40.4 × 6, T40.411, T40.412, T40.413, T40.414, T40.415, T40.416, T40.421, T40.422, T40.423, T40.424, T40.425, T40.426, T40.491, T40.492, T40.493, T40.494, T40.495, T40.496	No mention of fatal overdoses	Did not mention	A; S; D
***ICD-9, ICD-10***							
Chang^d^^S42^	2019	United States	2015–2016	965.01, E850.0, 965.00, 965.09, E850.2; T40.1, T40.3×, T40.0×, T40.2×, T40.4, T40.601×, T40.604×, T40.691×, T40.694×	No	Did not mention	
Chang^d^^S41^	2020	United States	2015–2016	965.01, E850.0, 965.00, 965.09, E850.2; T40.1, T40.3×, T40.0×, T40.2×, T40.4, T40.601×, T40.604×, T40.691×, T40.694×	No	Did not mention	
***ICD-9-CM, ICD-10-CM***							
Casillas^c^^S12^	2024	United States	2010–2020	T40.4 × 1, T40.4 × 2, T40.4 × 3, T40.4 × 4, T40.411, T40.412, T40.413, T40.414, T40.421, T40.422, T40.423, T40.424, T40.491, T40.492, T40.493, T40.494	No	Did not mention	A; S; D

ICD: International Classification of Diseases; ICD-9-CM: International Classification of Diseases, Ninth Edition, Clinical Modification; ICD-10-CM: International Classification of Diseases, Tenth Edition, Clinical Modification.

a Illicit opioids; ^b^Methadone and other opioids; ^c^Synthetic opioids; ^d^Heroin, methadone, and other opioids.

*Non-heroin opioid overdoses*. Some studies (*n* = 16) opted to define a subset of opioid overdoses as non-heroin opioid overdoses (Table 7).^S3,4,6,8-10,15,17,19,24,27,33,44–47^ Most studies (*n* = 11) used ICD-9-CM codes,^S3,6,10,17,19,27,33,44–47^ followed by ICD-10-CM codes (*n* = 8).^S3,4,6,8,10,15,45,47^ Few used ICD-10^S9,24,47^ or ICD-9^S9,24^ codes. Of studies applying ICD-9 or ICD-9-CM codes, all used 965.00 (opium) and 965.02 (methadone); most also used 965.09 (other opiates), E850.1 (methadone), E850.2 (other opiates) (Table 8). Many of the studies reporting ICD-10 and ICD-10-CM codes used T40.0×[1–4], T40.2×[1–4], T40.3×[1–4], T40.4×[1–4], T40.60[1–4], and T40.69[1–4] (Table 8). These codes were often used in concert, comprising a case definition applied across six of the eight studies that applied ICD-10-CM codes (Table 7).

**Table 7:** Summary of studies that defined a non-heroin opioid overdose (n = 16), which included studies published between 2013 and 2024 in the United States and Canada.

Author	Year	Country	Study period	Codes to define a non-heroin overdose	Combine fatal and nonfatal?	Case fields used (e.g., primary, secondary)	ICD-10-CM only: encounter type
***ICD-9-CM***							
Alinsky^S19^	2020	United States	2009–2015	E850.1, E850.2, 965.00, 965.02, 965.09	No mention of fatal overdoses	Did not mention	
Averitt^S44^	2020	United States	2006–2015	965.09, 965.00, 965.02	No mention of fatal overdoses	Did not mention	
Guy^S27^	2018	United States	2010–2014	965.00, 965.02, 965.09, E850.1, E850.2	No mention of fatal overdoses	Primary only	
Li^S46^	2020	United States	Jan 2005 - Sept 2015	965.00, 965.02, 965.09, E850.1, E850.2	No mention of fatal overdoses	Did not mention	
Mair^S33^	2018	United States	2004–2014	965.00, 965.02, 965.09, E850.1, E850.2	No mention of fatal overdoses	Other set number (e.g., first 5 positions)	
Peters^S17^	2018	United States	1998–2013	965.00, 965.02, 965.09, E850.1, E850.2	No mention of fatal overdoses	Did not mention	
***ICD-10-CM***							
Krishnaswami^S8^	2022	United States	Jan 2016 - Dec 2017	T40.0 × 1, T40.0 × 2, T40.0 × 3, T40.0 × 4, T40.2 × 1, T40.2 × 2, T40.2 × 3, T40.2 × 4, T40.3 × 1, T40.3 × 2, T40.3 × 3, T40.3 × 4, T40.4 × 1, T40.4 × 2, T40.4 × 3, T40.4 × 4, T40.601, T40.602, T40.603, T40.604, T40.691, T40.692, T40.693, T40.694	No	Any code field	A
Slavova^S15^	2020	United States	Oct 2015 - Dec 2016	T40.0 × 1, T40.0 × 2, T40.0 × 3, T40.0 × 4, T40.2 × 1, T40.2 × 2, T40.2 × 3, T40.2 × 4, T40.3 × 1, T40.3 × 2, T40.3 × 3, T40.3 × 4, T40.4 × 1, T40.4 × 2, T40.4 × 3, T40.4 × 4, T40.601, T40.602, T40.603, T40.604, T40.691, T40.692, T40.693, T40.694	No mention of fatal overdoses	Any code field	A; D
***ICD-9, ICD-10***							
Hartung^S24^	2020	United States	2015–2017	965.00, 965.02, 965.09, E850.1, E850.2, T40.0, T40.2, T40.3	Yes	Any code field	
Hartung^S9^	2022	United States	Jul 2014 - Dec 2017	965.00, 965.02, 965.09, E850.1, E850.2, T40.0×, T40.2×, T40.3×, T40.4×	No mention of fatal overdoses	Did not mention	
***ICD-9, ICD-10-CM***							
Chen^a^^S47^	2025	United States	2003–2020	965.00, 965.02, 965.09, E850.1, E850.2, T40.0 × 1, T40.2 × 1, T40.3 × 1, T40.4 × 1, T40.601, T40.691	No mention of fatal overdoses	Did not mention	A
***ICD-9-CM, ICD-10-CM***							
Chen^S10^	2021	United States	2010–2018	965.00, 965.02, 965.09, 970.1, E850.1, E850.2, T40.0 × 1, T40.0 × 2, T40.0 × 3, T40.0 × 4, T40.2 × 1, T40.2 × 2, T40.2 × 3, T40.2 × 4, T40.3 × 1, T40.3 × 2, T40.3 × 3, T40.3 × 4, T40.4 × 1, T40.4 × 2, T40.4 × 3, T40.4 × 4, T40.601, T40.602, T40.603, T40.604, T40.691, T40.692, T40.693, T40.694	No mention of fatal overdoses	Any code field	A; S; D
Chua^S45^	2020	United States	Jul 1, 2009 - Oct 1, 2017	965.00, 965.02, 965.09, E850.1, E850.2, T40.0 × 1, T40.0 × 2, T40.0 × 3, T40.0 × 4, T40.2 × 1, T40.2 × 2, T40.2 × 3, T40.2 × 4, T40.3 × 1, T40.3 × 2, T40.3 × 3, T40.3 × 4, T40.4 × 1, T40.4 × 2, T40.4 × 3, T40.4 × 4, T40.601, T40.602, T40.603, T40.604, T40.691, T40.692, T40.693, T40.694	No mention of fatal overdoses	Any code field	No specification
Coffey^b^^S4^	2020	United States	2012–2016	T40.0 × 1, T40.0 × 4, T40.2 × 1, T40.2 × 4, T40.3 × 1, T40.3 × 4, T40.4 × 1, T40.4 × 4, T40.601, T40.604, T40.691, T40.694	No mention of fatal overdoses	Other set number (e.g., first 5 positions)	A
Krishnaswami^S3^	2020	United States	2013–2016	965.00, 965.02, 965.09, E850.1, E850.2, T40.0 × 1, T40.0 × 2, T40.0 × 3, T40.0 × 4, T40.2 × 1, T40.2 × 2, T40.2 × 3, T40.2 × 4, T40.3 × 1, T40.3 × 2, T40.3 × 3, T40.3 × 4, T40.4 × 1, T40.4 × 2, T40.4 × 3, T40.4 × 4, T40.601, T40.602, T40.603, T40.604, T40.691, T40.692, T40.693, T40.694	No	Other set number (e.g., first 5 positions)^c^	A
Mayberry^S6^	2021	United States	2012–2016	965.00, 965.02, E850.1, 965.09, E850.2, T40.0 × 1, T40.0 × 2, T40.0 × 3, T40.0 × 4, T40.2 × 1, T40.2 × 2, T40.2 × 3, T40.2 × 4, T40.3 × 1, T40.3 × 2, T40.3 × 3, T40.3 × 4, T40.4 × 1, T40.4 × 2, T40.4 × 3, T40.4 × 4, T40.601, T40.602, T40.603, T40.604, T40.691, T40.692, T40.693, T40.694	No mention of fatal overdoses	Did not mention	A

ICD: International Classification of Diseases; ICD-9-CM: International Classification of Diseases, Ninth Edition, Clinical Modification; ICD-10-CM: International Classification of Diseases, Tenth Edition, Clinical Modification.

a Reported use of ICD-9/10 codes but reported codes consistent with ICD-10-CM.

b ICD-9-CM codes not listed.

c First listed diagnosis for ICD-9-CM codes and any code field for ICD-10-CM codes.

**Table 8:** Prevalence of ICD codes used to define nonfatal non-heroin overdoses (*n* = 16) identified in this review, which included studies published between 2013 and 2024 in the United States and Canada.

ICD version	ICD code	Definition	Prevalence
ICD-9	965.00	Poisoning by opium, unspecified	100%
	965.02	Poisoning by methadone	100%
	965.09	Poisoning by other opiates and related narcotics	100%
	E850.1	Accidental poisoning by methadone	100%
	E850.2	Accidental poisoning by other opiates and related narcotics	100%
ICD-9-CM	965.00	Poisoning by opium, unspecified	100%
	965.02	Poisoning by methadone	100%
	965.09	Poisoning by other opiates and related narcotics	91%
	E850.1	Accidental poisoning by methadone	91%
	E850.2	Accidental poisoning by other opiates and related narcotics	91%
	965.0	Poisoning by opiates and related narcotics	9%
	970.1	Poisoning by opiate antagonists	9%
ICD-10	T40.0	Poisoning by, adverse effect of and underdosing of opium	100%
	T40.2	Poisoning by, adverse effect of and underdosing of other opioids	100%
	T40.3	Poisoning by, adverse effect of and underdosing of methadone	100%
	T40.4	Poisoning by, adverse effect of and underdosing of other synthetic narcotics	100%
ICD-10-CM	T40.0 × 1	Poisoning by opium, accidental (unintentional)	100%
	T40.2 × 1	Poisoning by other opioids, accidental (unintentional)	100%
	T40.3 × 1	Poisoning by methadone, accidental (unintentional)	100%
	T40.4 × 1	Poisoning by, adverse effect of and underdosing of other synthetic narcotics (unintentional)	100%
	T40.601	Poisoning by unspecified narcotics, accidental (unintentional)	100%
	T40.691	Poisoning by other narcotics, accidental (unintentional)	100%
	T40.0 × 4	Poisoning by opium, undetermined	88%
	T40.2 × 4	Poisoning by other opioids, undetermined	88%
	T40.3 × 4	Poisoning by methadone, undetermined	88%
	T40.4 × 4	Poisoning by, adverse effect of and underdosing of other synthetic narcotics, undetermined	88%
	T40.604	Poisoning by unspecified narcotics, undetermined	88%
	T40.694	Poisoning by other narcotics, undetermined	88%
	T40.0 × 2	Poisoning by opium, intentional self-harm	75%
	T40.0 × 3	Poisoning by opium, assault	75%
	T40.2 × 2	Poisoning by other opioids, intentional self-harm	75%
	T40.2 × 3	Poisoning by other opioids, assault	75%
	T40.3 × 2	Poisoning by methadone, intentional self-harm	75%
	T40.3 × 3	Poisoning by methadone, assault	75%
	T40.4 × 2	Poisoning by, adverse effect of and underdosing of other synthetic narcotics, intentional self-harm	75%
	T40.4 × 3	Poisoning by, adverse effect of and underdosing of other synthetic narcotics, assault	75%
	T40.602	Poisoning by unspecified narcotics, intentional self-harm	75%
	T40.603	Poisoning by unspecified narcotics, assault	75%
	T40.692	Poisoning by other narcotics, intentional self-harm	75%
	T40.693	Poisoning by other narcotics, assault	75%

ICD: International Classification of Diseases; ICD-9-CM: International Classification of Diseases, Ninth Edition, Clinical Modification; ICD-10-CM: International Classification of Diseases, Tenth Edition, Clinical Modification.

Note: ICD code definitions from icd9data.com and icd10data.com

Of the studies reporting ICD-10-CM codes, the most common method involved specifying initial encounters only;^S3,4,6,8,47^ one study included initial and subsequent,^S15^ one study included initial, subsequent, and sequelae encounters,^S10^ and one study did not specify.^S45^ All eight studies included the unintentional intent specification – nearly all (*n* = 7) included undetermined intent, and both intentional and assault specifications were also common (*n* = 6).

Studies frequently did not include mention of how many case fields were used^S6,9,17,19,44,46,47^ or described searching across all case fields.^S8,10,15,24,45^ Only one study used primary case fields only;^S27^ the remaining three studies employed various approaches, such as searching differing case fields in specific periods or by ICD version.^S3,4,33^ Codes used to define non-heroin overdoses could include codes that other studies applied in measuring prescription opioid overdoses and other opioid overdose subclasses.

## 4 Discussion

This scoping review identified nuance in how nonfatal opioid overdoses are defined. The findings revealed inconsistencies in diagnostic codes applied, reflecting both uncertainty in the interpretation of ICD codes and an absence of a standardized operational definition for nonfatal opioid overdoses. These inconsistencies complicate public health monitoring efforts, hinder cross-study comparability, and may contribute to misclassification, which could result in either an undercount or overestimation of nonfatal opioid overdoses in epidemiological research. By synthesizing the myriad ways in which nonfatal opioid overdoses are defined, this review provides a foundation for advancing clarity and consistency in overdose monitoring and highlights the need for the development of standardized guidelines. These guidelines would need to be updated regularly, in tandem with updates to the ICD coding system. Below, we highlight specific recommendations for future research.

Studies applied differing ICD codes to define opioid overdoses. The proportion of studies using similar diagnostic codes varied by type of opioid. There appeared to be more congruence when considering ICD-9 and ICD-9-CM codes; for example, all studies included at least one diagnostic code (965.01) to define heroin overdoses (Table 3). Given that ICD-9 codes have been in use for a greater period of time than ICD-10 codes (Canadian Institute for Health Information (CIHI), 2025a; Centers for Medicare and Medicaid Services (CMS), 2025), researchers have had time to develop a consistent definition. Inconsistent definitions using ICD-10 codes could be attributable to facets of the ICD-10 coding system discussed in greater detail below. However, studies varied in diagnostic code inclusion for specific opioid types; for example, studies defining prescription opioids often contained codes for poisoning by opium. Additionally, studies varied in diagnostic code inclusion for opioid overdose symptoms (e.g., respiratory depression); a validation study supports the inclusion of diagnostic codes related to opioid overdose-specific symptoms (Mountcastle et al., 2019), though this was not commonly identified. Future research should consider conducting validation studies to move towards a consensus definition of nonfatal opioid overdose, with definitions created for clinically relevant sub-classes of opioid overdoses. Additional explorations with clinical experts, such as applying a Delphi methodological approach including a panel of clinical experts in addiction medicine, would be beneficial to understand clinically relevant diagnostic codes to include in definitions of nonfatal opioid overdoses (Nasa et al., 2021).

Second, specific attributes of ICD-10-CM codes varied. Intent specification was not consistently applied. Studies commonly included unintentional and undetermined intent, though studies also frequently applied intent and assault specifications. This is particularly salient as, if intent specifications are accurate, the mechanisms of action between an unintentional overdose and an intentional overdose may lead to differing interventions. Accurate conceptualization and measurement of intent is necessary. Intentional opioid overdoses are uniquely challenging to identify using claims data; validation studies reported substantial misclassification of intentional overdoses as unintentional^S172^ with other authors suggesting enhancement of algorithms via natural language processing.^S80^ Researchers should consider the complexity involved when including intent specifications and determine whether the mechanism of action specific to their study would operate differently by intent.

Third, studies varied by specified encounter type. It was common for studies not to specify types of encounters or to specify only initial encounters. This is consistent with guidance publicly available from the Council of State and Territorial Epidemiologists, which recommended including diagnostic codes with the 7th character missing or coded as an initial encounter.^14,S15^ Where possible, authors who intentionally include missing encounter diagnostic codes should specify this decision to improve transparency. Variation observed in this review could be attributable to changes in recommendations concerning the inclusion of encounter types over time.^S15^

Fourth, studies varied by reported case fields searched and how many were considered. As with other data elements, this could be an artifact of data structures. Authors should report data structures, including case fields in their data. Among the studies that did specify case fields searched, various definitions were applied (e.g., primary only vs. any position), which impacts the number of identified nonfatal overdoses: restricting inclusion to overdose diagnostic codes in primary case fields would exclude overdose cases coded in secondary positions. Results from validation studies suggest that expanding definitions to encompass all diagnoses (i.e., primary and secondary diagnoses) would return a high positivity of true overdose cases.^S15,210^ Future research should include clear reporting with justified decision-making regarding case fields, as recommended by several validation studies.^S15,210^

Fifth, while our study period aligned with the onset of increases in synthetic opioid overdose deaths (Centers for Disease Control and Prevention, 2025), we identified only one study that defined nonfatal fentanyl overdoses in isolation.^S43^ This could potentially be attributable to the introduction of a fentanyl-specific ICD-10 code in October 2020, for fiscal year 2021 (Centers for Medicare and Medicaid Services (CMS), 2020),^S43^ which was close to the end of our study period (October 2024), particularly when considering potential lags in data systems. Development of a working definition for nonfatal fentanyl overdoses could enable improved monitoring of regional and temporal trends across the United States and Canada.

Lastly, it was often unclear if discrepancies in definitions were intentional. Intentional discrepancies could result from the availability of certain types of diagnostic codes in the data. Data completeness will impact the overdose definition applied, particularly across healthcare settings;^S74^ data sharing agreements may limit diagnostic codes available by jurisdiction, further complicating consistent definitions.^S119^ Authors should consider reporting intended definitions alongside the diagnostic codes present in the data. In the absence of doing so, it is unclear whether specific ICD codes were not considered or were not available.

There are limitations inherent to this study. This review is not geared towards addressing public health monitoring complications arising from layperson-attended nonfatal overdoses. Variation in nonfatal overdose rates may arise due to differing naloxone dissemination or availability, which can impose variations across jurisdictions in terms of non-medically attended nonfatal overdoses. This review is focused on accurately defining medically attended nonfatal overdoses; future research should carefully consider methods for capturing non-medically attended nonfatal overdoses. Relatedly, a limitation of this review involves excluding 911 calls or overdoses solely attended to by paramedics, as this review focuses on exploring differences in nonfatal overdose definitions using administrative health data. Reports or grey literature not available from the searched databases are not included in this study. We are confident that our search returned a fair representation of studies, given the number of databases included and the number of manuscripts screened. Lastly, this study did not compare definitions from the peer-reviewed literature to those recommended by federal and state sources, such as the United States Centers for Disease Control and Prevention. Future research is warranted to amass definitions of nonfatal overdoses from such sources and compare recommended definitions to those prevalent in the peer-reviewed literature.

## 5 Conclusions

We found substantial variation in definitions of nonfatal opioid overdoses across studies. Key sources of variation included the actual diagnostic codes used, regardless of the ICD version applied, and case fields searched. Among studies using ICD-10-CM codes, variation included intent specifications and encounter types. Validation studies suggest avoiding the use of codes denoting adverse effects of opioids and including diagnostic codes across case fields. Differing data types and data sharing requirements across healthcare settings may impact the availability of diagnostic codes to construct a comprehensive opioid overdose definition. Detailed, standardized reporting methods when applying diagnostic codes to research would improve consistency and transparency.

## Research support

MEM is supported by NIH-NIDA R36DA061635 (PI: Marziali). RH and SSM were supported by NIH-NIDA R21DA057058 (PI: Hogg). SSM is also supported by NIH-NIDA R01DA059376 (PI: Martins). KYX was supported by NIH-NIDA K08DA061258. EMA is supported by NIH-NIDA T32DA031099 (MPI: Hasin, Martins).

## Relationships

There are no additional relationships to disclose.

## Patents and Intellectual Property

There are no patents to disclose.

## Other activities

There are no additional activities to disclose.

## CRediT authorship contribution statement

**Megan E. Marziali:** Writing – review & editing, Writing – original draft, Methodology, Investigation, Formal analysis, Conceptualization. **Silke Hansen:** Writing – review & editing, Writing – original draft, Investigation. **Kevin Y. Xu:** Writing – review & editing, Supervision, Methodology. **Esther Rowan:** Writing – review & editing, Writing – original draft, Investigation. **Michelle Doering:** Writing – review & editing, Resources, Methodology, Investigation. **Erin M. Annunziato:** Writing – review & editing, Writing – original draft, Investigation. **Robert S. Hogg:** Writing – review & editing, Supervision, Conceptualization. **Silvia S. Martins:** Writing – review & editing, Supervision, Conceptualization.

## Declaration of competing interest

The authors declare the following financial interests/personal relationships which may be considered as potential competing interests: MEM is supported by NIH-NIDA R36DA061635 (PI: Marziali). RH and SSM were supported by NIH-NIDA R21DA057058 (PI: Hogg). SSM is also supported by NIH-NIDA R01DA059376 (PI: Martins). KYX was supported by NIH-NIDA K08 DA061258. EMA is supported by NIH-NIDA T32DA031099 (MPI: Hasin, Martins). The authors declare no other financial or non-financial competing interests.

## Data Availability

Data will be made available on request.
