# Supplementary material for: Defining nonfatal opioid overdoses using administrative health data in the United States and Canada: A scoping review
Source: Prev Med Rep. 2026 Jul 31;69:103594. doi: 10.1016/j.pmedr.2026.103594 (PMC13474383; doi:10.1016/j.pmedr.2026.103594)
Supplement: Supplementary file 2 [file mmc1.docx]

**SUPPLEMENTAL.**

**Supplemental Table 1.** Example of the search in PubMed applied throughout this review.

| **Category of terms** | **Search terms** |
| --- | --- |
| (1) Drug overdose | Overdose*[tiab] OR Overdosing[tiab] OR Over-dose*[tiab] OR drug-dose*[tiab] OR medication-poison*[tiab] OR "drug overdose"[Mesh] |
| (2) Hospital-related | "International Classification of Diseases"[mesh] OR  International Classification of Disease*[tiab] OR  International Statistical Classification of Disease*[tiab] OR  ICD[tiab] OR  Diagnosis-code*[tiab] OR  diagnostic-code*[tiab] OR  "Electronic Health Records"[Mesh] OR electronic health record*[tiab] OR electronic medical record*[tiab] OR Computerized Medical Record*[tiab] OR EHR[tiab] OR  "Population Surveillance"[Mesh] OR  "Data Collection"[Mesh:NoExp] OR  "Data Accuracy"[Mesh] OR  "Datasets as Topic"[Mesh] OR  "Preliminary Data"[Mesh] OR  "Records"[Mesh:NoExp] OR  "Hospital Records"[Mesh] OR  "Medical Records"[Mesh] OR  "Morbidity"[Mesh:NoExp] OR  Incidence[mesh] OR  Prevalence[mesh] OR  "Registries"[Mesh] OR  "Data Aggregation"[Mesh] OR  standardized-data[tiab] OR  data-registr*[tiab] OR  data-repositor*[tiab] OR  data-set*[tiab] OR  data-source*[tiab] OR  data-system*[tiab] OR  surveillance[tiab] OR  discharge-data[tiab] OR  syndromic-data[tiab] OR  admission*-data[tiab] OR  hospital*-data[tiab] OR  ambulance-data[tiab] OR  paramedic-data[tiab] OR  emergency-department-data[tiab] OR  emergency-room-data[tiab] OR  aggregated-data[tiab] OR  data-coding[tiab] OR  data-codes OR  coded-data[tiab] OR  registries[tiab] OR  registry[tiab] OR  "MMWR Morb Mortal Wkly Rep"[Journal] OR  Clinical Emergency Data Registr*[tiab] OR  "Drug Overdose/classification"[Mesh]  "Emergency Medical Services/statistics and numerical data"[Mesh] OR  "Hospitalization/statistics and numerical data"[Mesh] OR  Clinical Emergency Department Registr*[tiab] OR CEDR[tiab] |

Note: Final search involved running search #1 and search #2 combined with NOT "Case Reports"[Publication Type]) AND ((english[Filter]) AND (2013:2024[pdat]))

**Supplemental Table 2.** Data charting elements applied when extracting data.

| **Data element** | **Type of response** |
| --- | --- |
| Title | Open-ended response |
| Lead author | Open-ended response |
| Year | Open-ended response |
| Country in which study was conducted  United States  Canada | Select all that apply |
| ***Characteristics of included studies*** | |
| Geography  National  State/province-specific | Multiple choice |
| What type of data?  ED  Inpatient (admissions)  Outpatient  Claims data  EMS  Other (open text) | Select all that apply |
| Study period | Open-ended response |
| ***Defining nonfatal overdoses*** | |
| Operationalization of nonfatal overdose  Exposure  Outcome  Study population | Multiple choice |
| Data to define nonfatal overdoses  Manual chart abstraction (e.g., “chief  complaint”)  Prescribed naloxone (e.g., “charge code”)  ICD-9  ICD-9-CM  ICD-10  ICD-10-CM  ICD-10-CA  SNOMED  Other (open text) | Select all that apply |
| List the ICD-9 codes included (if applicable) | Open-ended response |
| List the ICD-9-CM codes included (if applicable) | Open-ended response |
| List the ICD-10 codes included (if applicable) | Open-ended response |
| List the ICD-10-CM codes included (if applicable) | Open-ended response |
| List the ICD-10-CA codes included (if applicable) | Open-ended response |
| Do the authors combine nonfatal and fatal overdoses?  Yes  No  No mention of fatal overdoses | Multiple choice |
| If yes, what is the reason given for grouping fatal and nonfatal overdoses?  No reason provided  Sample size  Other (open text) | Multiple choice |
| If the authors restrict to specific drugs (e.g., “opioid-involved overdose”), what drugs are considered in the definition of nonfatal overdose?  Opioids  Stimulants  Benzodiazepines  No restriction  Other (open text) | Multiple choice |
| If the authors restrict to opioids, which categories do they use?  Any opioid  Heroin  Prescription opioids  Non-heroin opioids  Other (open text) | Select all that apply |
| ICD codes for any opioid | Open-ended response |
| ICD codes for heroin | Open-ended response |
| ICD codes for prescription opioids | Open-ended response |
| ICD codes for non-heroin opioids | Open-ended response |
| How many case fields were considered for diagnoses (e.g., opioid overdose code in the first X code fields)?  Primary only  Any code field  Other set number (e.g., first 5 positions)  Did not mention  Other (open text) | Multiple choice |
| Did the authors specify the final letter (A, S, D) for ICD-10-CM codes?  A  S  D  No specification | Select all that apply |
| Did the authors specify a window of inclusion for counting ICD codes as a single event?  1 day  2 days  7 days  30 days  No window specified | Select all that apply |
| Was this a validation study?  Yes  No | Multiple choice |
| Notes | Open-ended response |

**Supplemental Table 3.** Summary of studies that defined any opioid overdose (n=175) identified in this review, which included studies published between 2013-2024 in the United States and Canada.

| **Author** | **Year** | **Country** | **Study Period** | **Codes to define any opioid overdose** | **Combine fatal and nonfatal?** | **Case fields used (e.g., primary, secondary)** | **ICD-10-CM only: encounter type** |
| --- | --- | --- | --- | --- | --- | --- | --- |
| ***ICD-9*** | | | | | | | |
| Bagley^S49^ | 2020 | United States | Jan 1, 2012 - Dec 31, 2014 | 965.00, 965.01, 965.02, 965.09, E850.0, E850.1 E850.2 | No | Did not mention |  |
| Boscarino^S50^ | 2016 | United States | Apr 1, 2005 - Mar 31, 2015 | 965.00, 965.01, 965.02, 965.09, E850.0, E850.1, E850.2, E935.0, E935.1, E935.2 | No mention of fatal overdoses | Did not mention |  |
| Cochran^S51^ | 2017 | United States | 2007 - 2012 | 965.00, 965.02, 965.09, E850.1, E850.2 | Yes | Did not mention |  |
| Cochran^S52^ | 2017 | United States | 2010 - 2012 | 965.00, 965.02, 965.09, E850.1, E850.2 | Yes | Did not mention |  |
| Coupet^S53^ | 2020 | United States | 2009 - 2013 | 965.00, 965.01, 965.02, 965.09 | No mention of fatal overdoses | Did not mention |  |
| Fonda^S54^ | 2020 | United States | Apr 2007 - Sept 2012 | E850, E851, E852, E853, E854, E855, E856, E857, E858, E950.0, E950.1, E950.2, E950.3, E950.4, E950.5, E950.6, E980.0, 960, 961, 962, 963, 964, 965, 966, 967, 968, 969, 970, 971, 972, 973, 974, 975, 976, 977, 978, 979, E935.0, E935.1, E935.2 | No mention of fatal overdoses | Did not mention |  |
| Frazier^S55^ | 2017 | United States | 2008 - 2013 | 965.00, 965.01, 965.02, 965.09, E850.1, E850.2 | No mention of fatal overdoses | Did not mention |  |
| Glanz^S56^ | 2018 | United States | Jan 1, 2006 - Sept 30, 2015 | 965.0, 965.00, 965.01, 965.02, 965.09, E850.0, E850.1, E850.2 | Yes | Did not mention |  |
| Green^S57^ | 2017 | United States | 2003-2013 | 965.00, 965.01, 965.02, 965.09, E850.0, E850.1, E850.2 | Yes | Did not mention |  |
| Grossman^S58^ | 2020 | United States | 2010 - 2015 | 965.00, 965.01, 965.02, 965.09, E850.0, E850.1, E850.2 | No mention of fatal overdoses | Did not mention |  |
| Kuo^S59^ | 2018 | United States | Jan 2013 - Dec 2015 | 965, E850.1, E950.0, E980.0, E935.0, E935.1, E935.2, 276.4, 292.1, 292.8, 486, 496, 518.81, 518.82, E950, E951, E952, E953, E954, E955, E956, E957, E958, E959 | No mention of fatal overdoses | Any code field |  |
| Maeng^S60^ | 2017 | United States | Apr 1, 2005 - Mar 31, 2015 | 965.00, 965.01, 965.02, 965.09, E850.0, E850.1, E850.2, E935.0, E935.1, E935.2 | No mention of fatal overdoses | Did not mention |  |
| Meara^S61^ | 2016 | United States | 2006 - 2012 | 965.0x, 965.00, 965.02, 965.09, E850.1, E850.2, E950.0, E980.0, 965.01, E850.0 | No | Any code field |  |
| Morgan^S62^ | 2020 | United States | 2013 - 2015 | 965.0x, E850.0, E850.1, E850.2 | Yes | Did not mention |  |
| Mudumbai^S63^ | 2019 | United States | 2011 | E850.0, E850.1, E850.2, E935.0, E935.1, E935.2, E980.0, 965.0x | Yes | Did not mention |  |
| Olfson^S64^ | 2018 | United States | 2001 - 2007 | 965.00, 965.01, 965.02, 965.09, E850.0, E850.1, E850.2 | No | Did not mention |  |
| Rose^S65^ | 2019 | United States | 2011 - 2015 | 965.00, 965.01, 965.02, 965.09, E850.0, E850.1, E850.2 | No | Did not mention |  |
| Shi^S66^ | 2019 | United States | 2012 - 2013 | 965.00, 965.01, 965.02, 965.09 | No mention of fatal overdoses | Primary only |  |
| Stevens^S67^ | 2017 | United States | Jan 1, 2009 - Sept 31, 2015 | 965.00, 965.09, E850.0, E850.2, 965.02, 965.01, E850.0 | No | Any code field |  |
| Sun^S68^ | 2018 | United States | 2014 | 965.0x, E850.1, E950.0, E980.0, E935.0, E935.1, E935.2, 276.4, 292.1, 292.81, 292.8x, 486, 496, 518.81, 518.82, 780.0x, 780.97, 786.03, 786.05, 786.09, 786.52, 799.0x, E950, E951, E952, E953, E954, E955, E956, E957, E958, E959 | No mention of fatal overdoses | Did not mention |  |
| Warfield^S69^ | 2019 | United States | 2008 - 2016 | 965.00, 965.01, 965.02, 965.09, E850.0, E850.1, E850.2 | No | Did not mention |  |
| Yamamoto^S70^ | 2019 | United States | 2014 | 965.00, 965.01, 965.02, 965.09, E850.0, E850.1, E850.2 | No mention of fatal overdoses | Other set number (e.g., first 5 positions) |  |
| Yarborough^S71^ | 2016 | United States | NA | 965.x, E850.x, X42 | Yes | Did not mention |  |
| ***ICD-9-CM*** | | | | | | | |
| Alexandridis^S72^ | 2018 | United States | Jan 2009 - Dec 2014 | 965.0, 965.00, 965.01, 965.02, 965.09, E850.0, E850.1, E850.2 | No | Did not mention |  |
| Alinsky^S19^ | 2020 | United States | 2009 - 2015 | E850.0, E850.1, E850.2, 965.00, 965.01, 965.02, 965.09 | No mention of fatal overdoses | Did not mention |  |
| Archibald^S73^ | 2022 | United States | 2010 - 2014 | 965.0, 965.00, 965.01, 965.02, 965.09, E850.0, E850.1, E850.2 | No mention of fatal overdoses | Did not mention |  |
| Binswanger^S74^ | 2019 | United States | Jun 1, 2013 - Apr 1, 2014 | 965.0, 965.00, 965.01, 965.02, 965.09, E850.0, E850.1, E850.2, 965.9, E950.0, E950.2, E950.3, E950.4, E950.5, E980.0, E980.2, E980.3, E980.4, E980.5 | No mention of fatal overdoses | Did not mention |  |
| Campbell^S75^ | 2018 | United States | 2011 - 2014 | 965.0, 965.00, 965.01, 965.02, 965.09, E850.0, E850.1, E850.2 | Yes | Did not mention |  |
| Doshi^S76^ | 2019 | United States | Jan 2005 - Sept 2015 | 965.00, 965.02, 965.09 | No mention of fatal overdoses | Did not mention |  |
| Durrance^S77^ | 2024 | United States | 2005 - 2014 | 965.0, 965.00, 965.01, 965.02, 965.09, E850.0, E850.1, E850.2 | No mention of fatal overdoses | Any code field |  |
| Evans^S78^ | 2020 | United States | 2010 - 2014 | 965.00, 965.01, 965.02, 965.09, 970.1, E850.0, E850.1, E850.2 | No mention of fatal overdoses | Did not mention |  |
| Goldman-Mellor^S79^ | 2020 | United States | 2009 - 2011 | E850.0, E850.1, E850.2, 965.00, 965.01, 965.02, 965.09 | No | Any code field |  |
| Green^S80^ | 2019 | United States | Jan 1, 2008 - Dec 31, 2014 | 965.00, 965.01, 965.02, 965.09, E850.0, E850.1, E850.2 | Yes | Did not mention |  |
| Hasegawa^S81^ | 2014 | United States | Jan 1, 2010 - Dec 31, 2011 | 965.0, 965.00, 965.01, 965.02, 965.09, E950, E962.0 | No mention of fatal overdoses | Other set number (e.g., first 5 positions) |  |
| Hernandez^S82^ | 2018 | United States | Jan 1, 2013 - Dec 31, 2014 | 965.0, 965.00, 965.02, 965.09, E850.1, E850.2, E950.0, E980.0, 965.1, E850.05 | Yes | Did not mention |  |
| Hoffman^S83^ | 2017 | United States | Jan 1, 2006 - Dec 31, 2010 | 965.01, 965.02, 965.09 | No | Did not mention |  |
| Hume^S84^ | 2017 | United States | 2012 | 965.0, E850.0, E850.1, E850.2, 304.00, 304.01, 304.02, 304.70, 304.71, 304.72, 305.50, 305.51, 305.52 | Yes | Primary only |  |
| Jasuja^S85^ | 2021 | United States | 2011 - 2015 | 965.00, 965.01, 965.02, 965.09, E850.0, E850.1, E850.2 | No | Did not mention |  |
| Khan^S86^ | 2019 | United States | Jan 1, 2004 - Sept 30, 2015 | 965.00, 965.02, 965.09, E850.1, E850.2, E935.1, E935.2, 518.81, 518.82, 786.03, 786.05, 786.09, 799.0x, 799.1x | No mention of fatal overdoses | Did not mention |  |
| Larochelle^S87^ | 2015 | United States | May 2000 - Dec 2012 | 965.00, 965.01, 965.02, 965.09, E850.0, E850.1, E850.2 | No mention of fatal overdoses | Did not mention |  |
| Larochelle^S88^ | 2018 | United States | 2011 - 2015 | 965.00, 965.01, 965.02, 965.09, E850.0, E850.1, E850.2 | No | Did not mention |  |
| Larochelle^S89^ | 2019 | United States | 2011 - 2015 | 965.00, 965.01, 965.02, 965.09, E850.0, E850.1, E850.2 | No | Did not mention |  |
| Li^S90^ | 2022 | United States | Jan 1, 2009 - Dec 31, 2010 | 965.00, 965.01, 965.02, 965.09, E850.0, E850.1, E850.2 | Yes | Did not mention |  |
| Mountcastle^S91^ | 2019 | United States | Jan 2014 - Aug 2015 | 965.00, 965.02, 965.09, E850.1, E850.2, E935.1, E935.2, 518.81, 786.03, 799.01, 799.02, 799.1, 780.0, 780.01, 780.02, 780.09 | No mention of fatal overdoses | Any code field |  |
| Nam^S92^ | 2020 | United States | 1999 - 2012 | 965.00, 965.01, 965.02, 965.09, E850.0, E850.1, E850.2 | No mention of fatal overdoses | Any code field |  |
| Nielsen^S93^ | 2020 | United States | 2012 - 2014 | 965.00, 965.01, 965.02, 965.09, E850.0, E850.1, E850.2 | Yes | Did not mention |  |
| Park^S94^ | 2020 | United States | Jan 2012 - Dec 2015 | 965.00, 965.01, 965.02, 965.09, E850.0, E850.1, E850.2 | No | Did not mention |  |
| Reardon^S95^ | 2016 | United States | Jul 2009 - Jun 2012 | 965.00, 965.01, 965.02, 965.09, E850.0, E850.1, E850.2 | No mention of fatal overdoses | Any code field |  |
| Rowe^S96^ | 2017 | United States | Jan 1, 2012 - Dec 31, 2014 | 965.00, 965.01, 965.02, 965.09, E850.2 | No mention of fatal overdoses | Did not mention |  |
| Sears^S30^ | 2019 | United States | 2001 - 2014 | 965.00, 965.02, 965.09, 965.01 | No mention of fatal overdoses | Primary only |  |
| Sun^S97^ | 2020 | United States | 2011 - 2015 | 965.00, 965.02, 965.09, 965.01 | No mention of fatal overdoses | Did not mention |  |
| Weiner^S98^ | 2020 | United States | Jul 1, 2011 - Sept 30, 2015 | 965.00, 965.01, 965.02, 965.09, E850.0, E850.1, E850.2 | No | Did not mention |  |
| Young^S99^ | 2019 | United States | 2006 - 2015 | 965.00, 965.01, 965.02, 965.09, E850.0, E850.1, E850.2 | Yes | Did not mention |  |
| ***ICD-10*** | | | | | | | |
| Bozinoff^S100^ | 2022 | Canada | Jan 1, 2013 - Jan 1, 2019 | T40.0, T40.1, T40.2, T40.3, T40.4, T40.6 | Yes | Did not mention |  |
| Coupet^S101^ | 2021 | United States | Feb 2017 - Jan 2019 | T40.0, T40.1, T40.2, T40.3, T40.4, T40.6 | No mention of fatal overdoses | Did not mention |  |
| El Ibrahimi^S16^ | 2023 | United States | Jan 1, 2018 - Dec 31, 2018 | T40.1, T40.2, T40.3, T40.4, T40.6 | No | Did not mention |  |
| Forbes^S102^ | 2021 | United States | 2016 - 2017 | T40.0, T40.1, T40.2, T40.3, T40.4, T40.6, F11.1, F11.2, F11.9, F19.1, F19.2, F19.9, O99.32, R41.82, I46.9 | No mention of fatal overdoses | Did not mention |  |
| Gomes^S103^ | 2018 | Canada | Apr 2015 - Mar 2016 | T40.0, T40.1, T40.2, T40.3, T40.4, T40.6 | No mention of fatal overdoses | Did not mention |  |
| Gomes^S104^ | 2020 | Canada | Jul 2013 - Mar 2016 | T40.0, T40.1, T40.2, T40.3, T40.4, T40.6 | Yes | Did not mention |  |
| Houser^S105^ | 2021 | Canada | 2011 - 2017 | T40.0, T40.1, T40.2, T40.3, T40.4, T40.6 | No mention of fatal overdoses | Did not mention |  |
| Lebin^S106^ | 2021 | United States | Oct 1, 2015 - Oct 1, 2018 | T40 | No mention of fatal overdoses | Did not mention |  |
| Moe^S107^ | 2021 | Canada | Jan 1, 2015 - Dec 31, 2017 | T40.1, T40.6 | No | Did not mention |  |
| Morrow^S108^ | 2019 | Canada | 2006 - 2015 | T40.0, T40.1, T40.2, T40.3, T40.4, T40.6 | No mention of fatal overdoses | Other set number (e.g., first 5 positions) |  |
| Nguyen^S109^ | 2024 | Canada | Jan 1, 2016 - Mar 31, 2022 | T40.0, T40.1, T40.2, T40.3, T40.4, T40.6 | No | Did not mention |  |
| O'Brien^S110^ | 2019 | Canada | May 1, 2016 - Apr 31, 2017 | T40.0, T40.1, T40.2, T40.3, T40.4, T40.6 | No | Primary diagnosis |  |
| Patel^S111^ | 2021 | United States | Jan 1, 2019 - Oct 31, 2020 | T40.0x, T40.1x, T40.2x, T40.3x, T40.4x, T40.6x | Yes | Did not mention |  |
| Rosenbaum^S112^ | 2021 | United States | Dec 14, 2019 - Mar 22, 2020 | T40.0, T40.1, T40.2, T40.3, T40.4, T40.6, F11.0, F11.1, F11.2, F11.3, F11.4, F11.5, F11.6, F11.7, F11.8, F11.9 | No mention of fatal overdoses | Did not mention |  |
| Sacco^S113^ | 2023 | United States | 2016 | T40.0, T40.1, T40.2, T40.3, T40.4, T40.6 | No mention of fatal overdoses | Other set number (e.g., first 5 positions) |  |
| Thao^S114^ | 2024 | United States | Jan 1, 2016 - Jun 30, 2022 | T40.x | No mention of fatal overdoses | Did not mention |  |
| Weiner^S115^ | 2024 | United States | Jan 2017 - Dec 2019 | T40.0, T40.1, T40.2, T40.3, T40.4, T40.6 | Yes | Did not mention |  |
| Welk^S116^ | 2020 | Canada | 2013 - 2016 | F11, F11.0, F11.1, F11.2, F11.3, F11.4, F11.5, F11.6, F11.7, F11.8, F11.9, P96.1, T40.0, T40.1, T40.2, T40.3, T40.4, T40.5, T40.6, Y45.0 | No mention of fatal overdoses | Did not mention |  |
| ***ICD-10-CM*** | | | | | | | |
| Abasilim^S117^ | 2024 | United States | Jan 1, 2016 - Dec 31, 2021 | F11.12, F11.22, F11.92, T40, T41, T42, T43, T44,  T46 | No | Did not mention | No specification |
| Boyer^S118^ | 2022 | United States | Jan 1, 2018 - Dec 31, 2018 | T40.0X1, T40.0X2, T40.0X3, T40.0X4, T40.1X1, T40.1X2, T40.1X3, T40.1X4, T40.3X1, T40.3X2, T40.3X3, T40.3X4, T40.2X1, T40.2X2, T40.2X3, T40.2X4, T40.4X1, T40.4X2, T40.4X3, T40.4X4 T40.601, T40.602, T40.603, T40.604, T40.691, T40.692, T40.693, T40.694 | No mention of fatal overdoses | Any code field | A |
| Brathwaite^S119^ | 2021 | United States | Jan 2018 - Jul 2018 | T40.1X1, T40.1X4, T40.0X1, T40.0X4, T40.2X1, T40.2X4, T40.3X1, T40.3X4, T40.4X1, T40.4X4, T40.601, T40.604, T40.691, T40.694, F11.12, F11.120, F11.121, F11.122, F11.129, F11.22, F11.220, F11.221, F11.222, F11.229, F11.92, F11.920, F11.921, F11.922, F11.929, F11.10, F11.20, F11.90 | No mention of fatal overdoses | Did not mention | A |
| Chen^S120^ | 2024 | United States | 2020 | T40.0X1, T40.0X2, T40.0X3, T40.0X4, T40.1X1, T40.1X2, T40.1X3, T40.1X4, T40.2X1, T40.2X2, T40.2X3, T40.2X4, T40.3X1, T40.3X2, T40.3X3, T40.3X4, T40.4X1, T40.4X2, T40.4X3, T40.4X4, T40.6X1, T40.6X2, T40.6X3, T40.6X4 | Yes | Any code field | A |
| Chua^S121^ | 2022 | United States | Aug 4, 2019 - Apr 3, 2021 | F11.12, F11.120, F11.121, F11.122, F11.129, F11.22, F11.220, F11.221, F11.222, F11.229, F11.92, F11.920, F11.921, F11.922, F11.929, T40.0X1, T40.0X2, T40.0X3, T40.0X4, T40.1, T40.1X, T40.1X1, T40.1X2, T40.1X3, T40.1X4, T40.2X1, T40.2X2, T40.2X3, T40.2X4, T40.3X1, T40.3X2, T40.3X3, T40.3X4, T40.411, T40.412, T40.413, T40.414, T40.421, T40.422, T40.423, T40.424, T40.491, T40.492, T40.493, T40.494, T40.4X1, T40.4X2, T40.4X3, T40.4X4, T40.601, T40.602, T40.603, T40.604, T40.691, T40.692, T40.693, T40.694 | No mention of fatal overdoses | Any code field | A; S; D |
| Chua^S122^ | 2021 | United States | January 1, 2017 to December 31, 2018 | T40.0X1, T40.0X2, T40.0X3, T40.0X4, T40.2X1, T40.2X2, T40.2X3, T40.2X4, T40.3X1, T40.3X2, T40.3X3, T40.3X4, T40.4X1, T40.4X2, T40.4X3, T40.4X4, T40.601, T40.602, T40.603, T40.604, T40.691, T40.692, T40.693, T40.694 | No mention of fatal overdoses | Any code field | No specification |
| Faude^S123a^ | 2023 | United States | Dec 2018 - Jul 2020 | T40.0, T40.1, T40.2, T40.3, T40.4, T40.6 | No mention of fatal overdoses | Did not mention | No specification |
| Fix^S124^ | 2021 | United States | Jan 1, 2017 - Nov 30, 2017 | T40.0X1, T40.0X2, T40.0X3, T40.0X4, T40.1X1, T40.1X2, T40.1X3, T40.1X4, T40.2X1, T40.2X2, T40.2X3, T40.2X4, T40.3X1, T40.3X2, T40.3X3, T40.3X4, T40.4X1, T40.4X2, T40.4X3, T40.4X4 | No mention of fatal overdoses | Any code field | A; D |
| Friedman^S125^ | 2025 | United States | Jan 1, 2016 - Dec 31, 2021 | F11.12, F11.22, F11.92, T40.0X1, T40.0X2, T40.0X3, T40.0X4, T40.1X1, T40.1X2, T40.1X3, T40.1X4, T40.2X1, T40.2X2, T40.2X3, T40.2X4, T40.3X1, T40.3X2, T40.3X3, T40.3X4, T40.4X1, T40.4X2, T40.4X3, T40.4X4, T40.601, T40.602, T40.603, T40.604, T40.691, T40.692, T40.693, T40.694 | No | Did not mention | A |
| Harder^S126^ | 2021 | United States | Jan 1, 2016 - Jun 30, 2018 | T40.0X1, T40.0X2, T40.0X3, T40.0X4, T40.1X1, T40.1X2, T40.1X3, T40.1X4, T40.2X1, T40.2X2, T40.2X3, T40.2X4, T40.3X1, T40.3X2, T40.3X3, T40.3X4, T40.4X1, T40.4X2, T40.4X3, T40.4X4, T40.601, T40.602, T40.603, T40.604, T40.691, T40.692, T40.693, T40.694, T50.901, T50.902, T50.903, T50.904 | No mention of fatal overdoses | Did not mention | A; S; D |
| Harder^S127^ | 2021 | United States | Oct 2015 - Dec 2018 | T40.0X1, T40.0X2, T40.0X3, T40.0X4, T40.1X1, T40.1X2, T40.1X3, T40.1X4, T40.2X1, T40.2X2, T40.2X3, T40.2X4, T40.3X1, T40.3X2, T40.3X3, T40.3X4, T40.4X1, T40.4X2, T40.4X3, T40.4X4, T40.601, T40.602, T40.603, T40.604, T40.691, T40.692, T40.693, T40.694, T50.901, T50.902, T50.903, T50.904 | Yes | Did not mention | A; S; D |
| Jones^S128^ | 2024 | United States | Jan 2020 - Dec 2021 | T40.0, T40.1, T40.2, T40.3, T40.4, T40.6 | No | Any code field | No specification |
| Liu^S129^ | 2020 | United States | Jan 1, 2018 - Dec 31, 2019 | T40.0X1, T40.0X4, T40.1X1, T40.1X4, T40.2X1, T40.2X4, T40.3X1, T40.3X4, T40.4X1, T40.4X4, T40.601, T40.604, T40.691, T40.694 | No mention of fatal overdoses | Did not mention | A |
| Lloyd^S130^ | 2024 | United States | 2018 - 2020 | T40.0X1, T40.0X2, T40.0X3, T40.0X4, T40.1X1, T40.1X2, T40.1X3, T40.1X4, T40.2X1, T40.2X2, T40.2X3, T40.2X4, T40.3X1, T40.3X2, T40.3X3, T40.3X4, T40.6X1, T40.6X2, T40.6X3, T40.6X4 | No mention of fatal overdoses | Did not mention | No specification |
| Mannes^S131^ | 2023 | United States | Mar 1, 2019 - Dec 31, 2020 | T40.0X1, T40.0X2, T40.0X3, T40.0X4, T40.1X1, T40.1X2, T40.1X3, T40.1X4, T40.3X1, T40.3X2, T40.3X3, T40.3X4, T40.411, T40.412, T40.413, T40.414, T40.423, T40.424, T40.491, T40.492, T40.493, T40.494, T40.4X1, T40.4X2, T40.4X3, T40.4X4, T40.601, T40.602, T40.603, T40.604, T40.691, T40.692, T40.693, T40.694, T40.2X1, T40.2X2, T40.2X3, T40.2X4 | Yes | Did not mention | A |
| Martin^S132^ | 2024 | United States | Jan 1, 2019 - Dec 31, 2020 | T40.0X1, T40.0X2, T40.0X3, T40.0X4, T40.1X1, T40.1X2, T40.1X3, T40.1X4, T40.2X1, T40.2X2, T40.2X3, T40.2X4, T40.3X1, T40.3X2, T40.3X3, T40.3X4, T40.4X1, T40.4X2, T40.4X3, T40.4X4, T40.601, T40.602, T40.603, T40.604, T40.691, T40.692, T40.693, T40.694, T40.411, T40.412, T40.413, T40.414, T40.421, T40.422, T40.423, T40.424. T40.491, T40.492, T40.493, T40.494 | No mention of fatal overdoses | Primary only | No specification |
| Moreland^S133^ | 2021 | United States | 2018 | T40.0X1, T40.1X1, T40.2X1, T40.3X1, T40.4X1, T40.601, T40.691 | No mention of fatal overdoses | Any code field | No specification |
| Peterson^S13^ | 2019 | United States | 2016 | T40.0X1, T40.0X2, T40.0X3, T40.0X4, T40.1X1, T40.1X2, T40.1X3, T40.1X4, T40.2X1, T40.2X2, T40.2X3, T40.2X4, T40.3X1, T40.3X2, T40.3X3, T40.3X4, T40.4X1, T40.4X2, T40.4X3, T40.4X4, T40.6X1, T40.6X2, T40.6X3, T40.6X4 | No mention of fatal overdoses | Other set number (e.g., first 5 positions) | No specification |
| Rock^S134^ | 2021 | United States | Jan 1, 2018 - Dec 31, 2019 | T40.0X1, T40.0X2, T40.0X3, T40.0X4, T40.1X1, T40.1X2, T40.1X3, T40.1X4, T40.2X1, T40.2X2, T40.2X3, T40.2X4, T40.3X1, T40.3X2, T40.3X3, T40.3X4, T40.4X1, T40.4X2, T40.4X3, T40.4X4, T40.601, T40.602, T40.603, T40.604, T40.691, T40.692, T40.693, T40.694 | No mention of fatal overdoses | Did not mention | A |
| Roth^S135^ | 2024 | United States | 2016 - 2019 | T40.0X1, T40.0X2, T40.0X3, T40.0X4, T40.1X1, T40.1X2, T40.1X3, T40.1X4, T40.2X1, T40.2X2, T40.2X3, T40.2X4, T40.3X1, T40.3X2, T40.3X3, T40.3X4, T40.4X1, T40.4X2, T40.4X3, T40.4X4, T40.601, T40.602, T40.603, T40.604, T40.691, T40.692, T40.693, T40.694 | No | Did not mention | A |
| Soares^S136^ | 2022 | United States | Jan 1, 2018 - Dec 31, 2020 | T40.0X, T40.1X, T40.2X, T40.3X, T40.4X, T40.6X | No mention of fatal overdoses | Did not mention | No specification |
| Sorensen^S137^ | 2023 | United States | Feb 2019 - Apr 2022 | T40.0X1, T40.0X2, T40.0X4, T40.1X1, T40.1X2, T40.1X4, T40.2X1, T40.2X2, T40.2X4, T40.3X1, T40.3X2, T40.3X4, T40.411, T40.412, T40.414, T40.421, T40.422, T40.424, T40.491, T40.492, T40.494, T40.601, T40.602, T40.604, T40.691, T40.692, T40.694 | No mention of fatal overdoses | Did not mention | A; S; D |
| Spencer^S138^ | 2023 | United States | 2016 | F11.92, F11.12, F11.22, T40.0, T40.1, T40.2, T40.3, T40.4, T40.6 | No | Did not mention | No specification |
| Stevens^S139^ | 2023 | United States | Mar 2018 - Feb 2022 | T40.0X1, T40.0X2, T40.0X3, T40.0X4, T40.0X5, T40.1X1, T40.1X2, T40.1X3, T40.1X4, T40.2X1, T40.2X2, T40.2X3, T40.2X4, T40.2X5, T40.3X1, T40.3X2, T40.3X3, T40.3X4, T40.3X5, T40.4X1, T40.4X2, T40.4X3, T40.4X4, T40.4X5 | No mention of fatal overdoses | Did not mention | A |
| Vivolo-Kantor^S140^ | 2018 | United States | Jul 2016 - Sept 2017 | T40.0, T40.1, T40.2, T40.3, T40.4, T40.6, T40.69, F11.12, F11.22, F11.92 | No mention of fatal overdoses | Did not mention | No specification |
| Vivolo-Kantor^S141^ | 2020 | United States | 2016 - 2017 | T40.0X1, T40.0X2, T40.0X3, T40.0X4, T40.1X1, T40.1X2, T40.1X3, T40.1X4, T40.2X1, T40.2X2, T40.2X3, T40.2X4, T40.3X1, T40.3X2, T40.3X3, T40.3X4, T40.4X1, T40.4X2, T40.4X3, T40.4X4, T40.601, T40.602, T40.603, T40.604, T40.691, T40.692, T40.693, T40.694 | No mention of fatal overdoses | Any code field | A |
| Weiner^S142^ | 2019 | United States | 2015 - 2018 | T40.0X1, T40.0X2, T40.0X5, T40.1X1, T40.1X2, T40.1X4, T40.2X1, T40.2X2, T40.2X4, T40.2X5, T40.3X1, T40.3X2, T40.3X4, T40.3X5, T40.4X1, T40.4X2, T40.4X4, T40.4X5, T40.601, T40.602, T40.603, T40.604, T40.605, T40.691, T40.695 | No mention of fatal overdoses | Did not mention | A; S; D |
| Weiner^S143^ | 2022 | United States | 2018 - 2021 | T40.0X1, T40.1X1, T40.1X2, T40.1X4, T40.2X1, T40.2X2, T40.2X4, T40.3X1, T40.3X2, T40.3X4, T40.411, T40.412, T40.491, T40.601, T40.602, T40.604, T40.691 | No mention of fatal overdoses | Did not mention | A; S; D |
| White^S144^ | 2021 | United States | 2016 | T40.0X1, T40.0X2, T40.0X3, T40.0X4, T40.1X1, T40.1X2, T40.1X3, T40.1X4, T40.2X1, T40.2X2, T40.2X3, T40.2X4, T40.3X1, T40.3X2, T40.3X3, T40.3X4, T40.4X1, T40.4X2, T40.4X3, T40.4X4, T40.601, T40.602, T40.603, T40.604, T40.691, T40.692, T40.693, T40.694, F11.92, F11.920, F11.921, F11.922, F11.929, F11.12, F11.120, F11.121, F11.122, F11.129, F11.22, F11.220, F11.221, F11.222, F11.229 | No mention of fatal overdoses | Any code field | A; S; D |
| ***ICD-10-CA*** |  |  |  |  |  |  |  |
| Carnide^S145^ | 2024 | Canada | 2006 - 2020 | T40.0, T40.1, T40.2, T40.20, T40.21, T40.22, T40.23, T40.28, T40.3, T40.4, T40.40, T40.41, T40.48, T40.6 | No mention of fatal overdoses | Did not mention |  |
| Leece^S146^ | 2019 | Canada | Jan 1, 2015 - Dec 31, 2016 | T40.0, T40.1, T40.2, T40.3, T40.4, T40.5, T40.6 | No | Did not mention |  |
| O'Connor^S147^ | 2018 | Canada | 2007 - 2017 | T40.0, T40.1, T40.2, T40.3, T40.4, T40.6 | No mention of fatal overdoses | Other: M (most responsible diagnosis), 1 (pre-admit comorbidity), 2 (post-admit comorbidity), 6 (proxy most-responsible diagnosis), W, X, Y (service transfer diagnoses) |  |
| Rudoler^S148^ | 2022 | Canada | Sept 1, 2011 - Aug 31, 2013 | T40.0, T40.1, T40.2, T40.3, T40.4, T40.6 | No mention of fatal overdoses | Did not mention |  |
| ***ICD-9, ICD-10*** | | | | | | | |
| Alobaidi^S149^ | 2021 | United States | 2009 - 2018 | 965.00, 965.01, 965.02, 965.09, E850.0, E850.1, E850.2, E950.0, E980.0, T40.1X, T40.2X, T40.4X, T40.3X, T40.69, T40.60 | No mention of fatal overdoses | Did not mention |  |
| Andersen^S150^ | 2022 | United States | 2010 - 2016 | 965.0, E850.1, E950.0, E980.0, E935.0, E935.1, E935.2, T40.0, T40.2, T40.3, T40.4, 276.4, 292.1, 292.8, 486, 496, 518.81, 518.82, 780.0x, 780.97, 786.03, 786.05, 786.09, 786.52, 799.0x, E950, E951, E952, E953, E954, E955, E956, E957, E958, E959 | No mention of fatal overdoses | Did not mention |  |
| Badger^S151^ | 2019 | United States | Jan 1999 - Jun 2016 | 965.01, E850.0, 965.00, 965.02, E850.1, 965.09, E850.2, T40.1, T40.0, T40.2, T40.3, T40.4, T40.6, T40.69 | No mention of fatal overdoses | Did not mention |  |
| Chang^S152^ | 2023 | United States | 2005 - 2019 | E850.1, E850.2, 965.0, E850.0, E935.0, E935.1, E935.2, T40.0, T40.1, T40.2, T40.3, T40.4, T40.6 | No mention of fatal overdoses | Other set number (e.g., first 5 positions) |  |
| Cicero^S153^ | 2019 | United States | Jul 1, 2014 - Jun 30, 2016 | 965.09, T40.2, T20.4, T40.6 | No mention of fatal overdoses | Did not mention |  |
| Daoust^S154^ | 2020 | Canada | Jan 1, 2004 - Mar 31, 2014 | 965.00, 965.80, T40.0, T40.1, T40.2, T40.3, T40.4, T40.6 | No mention of fatal overdoses | Did not mention |  |
| Gibbons^S155^ | 2022 | United States | 2010 - 2017 | 965.00, 965.01, 965.02, 965.09, E850.0, E850.1, E850.2, T40.0x, T40.1x, T40.2x, T40.3x, T40.4x | No mention of fatal overdoses | Did not mention |  |
| Hartung^S9^ | 2022 | United States | Jul 2014 - Dec 2017 | 965.00, 965.01, 965.02, 965.09, E850.0, E850.1, E850.2, T40.0x, T40.1x, T40.2x, T40.3x, T40.4x | No mention of fatal overdoses | Did not mention |  |
| Hendricks^S156^ | 2023 | United States | Jan 1, 2015 - Dec 31, 2018 | 965.00, 965.01, 965.02, 965.09, E850.0, E850.1, E850.2, T40.1, T40.2, T40.3, T40.4, T40.6 | Yes | Did not mention |  |
| Lindner^S157^ | 2023 | United States | Jan 1, 2016 - Dec 31, 2018 | 965.00, 965.01, 965.02, 965.09, E850.0, E850.1, E850.2, T40.0, T40.1, T40.2, T40.3, T40.4, X42, X62, Y12 | No mention of fatal overdoses | Did not mention |  |
| Nguyen^S158^ | 2023 | United States | Jan 2015 - Jun 2021 | 965.0, 965.00, 965.1, 965.02, 965.09, E850.0, E850.1, E850.2, T40.0, T40.1, T40.2, T40.3, T40.4, T40.6 | Yes | Did not mention |  |
| Qeadan^S159^ | 2022 | United States | Jan 1, 2009 - Dec 31, 2017 | 965.00, 965.01, 965.02, 965.09, E850.0, E850.1, E850.2, T40.0, T40.1, T40.2, T40.3, T40.4, T40.6 | No mention of fatal overdoses | Did not mention |  |
| Saloner^S160^ | 2020 | United States | 2015 | 965.00, 965.01, 965.02, 965.09, E850.0, E850.1, E850.2, T40.0x, T40.1x, T40.2x, T40.3x, T40.4 | No | Did not mention |  |
| Smolina^S161^ | 2019 | Canada | Jan 1, 2015 - Nov 30, 2016 | 965.0, E850.0, T40.0, T40.1, T40.2, T40.3, T40.4, T40.6 | Yes | Primary only |  |
| Suffoletto^S162^ | 2020 | United States | Jul 2015 - Jan 2018 | 956.0, E850, T40.0, X42, X62, Y12 | No | Did not mention |  |
| Sugarman^S163^ | 2024 | United States | Jul 1, 2016 - Dec 31, 2020 | 965.00, 965.01, 965.02, 965.09, E850.0, E850.1, E850.2, T40.0x, T40.1x, T40.2x, T40.3x, T40.4 | No | Did not mention |  |
| Sun^S164^ | 2022 | United States | Jan 1, 2010 - Dec 31, 2018 | 965.00, 965.01, 965.02, 965.09, E850.0, E850.1, E850.2, T40.0, T40.1, T40.2, T40.3, T40.6 | No mention of fatal overdoses | Did not mention |  |
| Weiner^S165^ | 2022 | United States | 2013 - 2018 | 965.00, 965.01, 965.02, 965.09, E850.0, E850.1, E850.2, T40.0, T40.1, T40.2, T40.3, T40.4, T40.6 | Yes | Did not mention |  |
| Weiner^S166^ | 2022 | United States | 2014 -2018 | 965.00, 965.01, 965.02, 965.09, E850.0, E850.1, E850.2, T40.0, T40.1, T40.2, T40.3, T40.4, T40.6 | Yes | Did not mention |  |
| Williams^S167^ | 2024 | United States | Apr 2011 - Mar 2018 | 965, 965.01, 965.02, 965.09, E850.0, E850.1, E850.2, T40.0, T40.1, T40.2, T40.3, T40.4 | No | Did not mention |  |
| Williams^S168^ | 2020 | United States | 2013 - 2017 | 965.0, E850.0, E850.1, E850.2, T40.0, T40.1, T40.2, T40.3, T40.4 | No mention of fatal overdoses | Did not mention |  |
| ***ICD-9-CM, ICD-10-CM*** | | | | | | | |
| Agnoli^S169^ | 2021 | United States | 2007 - 2019 | 965.00, 965.01, 965.02, 965.09, E850.0, E850.1, E850.2, T40.0X1, T40.0X2, T40.0X3, T40.0X4, T40.1X1, T40.1X2, T40.1X3, T40.1X4, T40.2X1, T40.2X2, T40.2X3, T40.2X4, T40.3X1, T40.3X2, T40.3X3, T40.3X4, T40.4X1, T40.4X2, T40.4X3, T40.4X4, T40.601, T40.602, T40.603, T40.604, T40.691, T40.692, T40.693, T40.694 | No | Did not mention | A |
| Arakkal^S170^ | 2024 | United States | 2001 - 2021 | 965.0, 965.00, 965.01, 965.02, 965.09, E850.0, E850.1, E850.2, T40.0, T40.0X, T40.0X1, T40.0X2, T40.0X3, T40.0X4, T40.0X5, T40.0X6, T40.1, T40.1X, T40.1X1, T40.1X2, T40.1X3, T40.1X4, T40.2, T40.2X, T40.2X1, T40.2X2, T40.2X3, T40.2X4, T40.2X5, T40.2X6, T40.3, T40.3X, T40.3X1, T40.3X2, T40.3X3, T40.3X4, T40.3X5, T40.3X6, T40.4, T40.4X, T40.4X1, T40.4X2, T40.4X3, T40.4X4, T40.4X5 | No mention of fatal overdoses | Did not mention | A; S; D |
| Banta-Green^S171^ | 2019 | United States | NA | 965.00, 965.01, 965.02, 965.09, E850.0, E850.1, E850.2, T40.0X1, T40.0X2, T40.0X3, T40.0X4, T40.1X1, T40.1X2, T40.1X3, T40.1X4, T40.2X1, T40.2X2, T40.2X3, T40.2X4, T40.3X1, T40.3X2, T40.3X3, T40.3X4, T40.4X1, T40.4X2, T40.4X3, T40.4X4 | No | Primary only | No specification |
| Beachler^S172^ | 2022 | United States | Jul 1, 2008 - Sept 30, 2015 | 965.00, 965.01, 965.02, 965.09, E850.0, E850.1, E850.2, 965.0, T40.0x, T40.1, T40.2x, T40.3x, T40.4x, X42, X62, Y12 | Yes | Any code field | A; S; D |
| Bovell-Ammon^S173^ | 2024 | United States | 2014 - 2020 | 965.00, 965.01, 965.02, 965.09, 970.1, E850.0, E850.1, E850.2, T40.0X1, T40.0X2, T40.0X3, T40.0X4, T40.1X1, T40.1X2, T40.1X3, T40.1X4, T40.2X1, T40.2X2, T40.2X3, T40.2X4, T40.3X1, T40.3X2, T40.3X3, T40.3X4, T40.4X1, T40.4X2, T40.4X3, T40.4X4, T40.601, T40.602, T40.603, T40.604, T40.691, T40.692, T40.693, T40.694 | Yes | Did not mention | A; D |
| Bykov^S174^ | 2020 | United States | Oct 1, 2007 - Dec 31, 2017 | 965.00, 965.02, 965.09, E850.1, E850.2, T40.0X1, T40.0X2, T40.0X3, T40.0X4, T40.2X1, T40.2X2, T40.2X3, T40.2X4, T40.3X1, T40.3X2, T40.3X3, T40.3X4, T40.4X1, T40.4X2, T40.4X3, T40.4X4, T40.601, T40.602, T40.603, T40.604, T40.691, T40.692, T40.693, T40.694 | No mention of fatal overdoses | Did not mention | A; S; D |
| Casillas^S12^ | 2024 | United States | 2010 - 2020 | 965.00, 965.01, 965.02, 965.09, E850.0, E850.1, E850.2, T40.0X1, T40.0X2, T40.0X3, T40.0X4, T40.1X1, T40.1X2, T40.1X3, T40.1X4, T40.2X1, T40.2X2, T40.2X3, T40.2X4, T40.3X1, T40.3X2, T40.3X3, T40.3X4, T40.4X1, T40.4X2, T40.4X3, T40.4X4, T40.411, T40.412, T40.413, T40.414, T40.421, T40.422, T40.423, T40.424, T40.491, T40.492, T40.493, T40.494, T40.601, T40.602, T40.603, T40.604, T40.691, T40.692, T40.693, T40.694 | No | Did not mention | A; S; D |
| Chang^S11^ | 2019 | United States | 2015 | 965.00, 965.01, 965.02, 965.09, 970.1, E850.0, E850.1, E850.2, T40.0X1, T40.0X4, T40.1X1, T40.1X4, T40.2X1, T40.2X4, T40.3X1, T40.3X4, T40.4X1, T40.4X4, T40.601, T40.604, T40.691, T40.694 | No mention of fatal overdoses | Other set number (e.g., first 5 positions) | A; S; D |
| Chang^S175^ | 2024 | United States | 2010 - 2019 | 965.00, 965.01, 965.02, 965.09, E850.0, E850.1, E850.2, E935.0, E935.1, E935.2, T40.0X1, T40.0X2, T40.0X3, T40.0X4, T40.1X1, T40.1X2, T40.1X3, T40.1X4, T40.3X1, T40.3X2, T40.3X3, T40.3X4, T40.2X1, T40.2X2, T40.2X3, T40.2X4, T40.4X1, T40.4X2, T40.4X3, T40.4X4, T40.601, T40.602, T40.603, T40.604, T40.691, T40.692, T40.693, T40.694 | No | Other set number (e.g., first 5 positions) | A |
| Chen^S176^ | 2021 | United States | 2010 - 2016 | 965.0, 965.00, 965.01, 965.02, 965.09, E850.0, E850.1, E850.2, T40.0X1, T40.0X2, T40.0X3, T40.0X4, T40.0X5, T40.1X1, T40.1X2, T40.1X3, T40.1X4, T40.2X1, T40.2X2, T40.2X3, T40.2X4, T40.2X5, T40.3X1, T40.3X2, T40.3X3, T40.3X4, T40.3X5, T40.4X1, T40.4X2, T40.4X3, T40.4X4, T40.4X5 | No mention of fatal overdoses | Other set number (e.g., first 5 positions) | A; S |
| Cherian^S177^ | 2024 | United States | May 1, 2011 - Dec 31, 2017 | 965.00, 965.01, 965.02, 9965.09, E850.0, E850.1, E850.2, T40.0X, T40.1X, T40.2X, T40.3X, T40.4X, T40.60, T40.69 | No mention of fatal overdoses | Any code field | No specification |
| Chiuve^S178^ | 2021 | United States | 2006 - 2017 | E850.0, E850.1, 965.0, 965.00, 965.01, 965.02, 965.09, T40.0, T40.0X, T40.0X1, T40.0X2, T40.0X3, T40.0X4, T40.0X5, T40.0X6 | No mention of fatal overdoses | Any code field | A; S; D |
| Chua^S179^ | 2021 | United States | 2011 - 2018 | 965.00, 965.02, 965.09, E850.1, E850.2, T40.0X1, T40.0X2, T40.0X3, T40.0X4, T40.2X1, T40.2X2, T40.2X3, T40.2X4, T40.3X1, T40.3X2, T40.3X3, T40.3X4, T40.4X1, T40.4X2, T40.4X3, T40.4X4, T40.601, T40.602, T40.603, T40.604, T40.691, T40.692, T40.693, T40.694 | No mention of fatal overdoses | Did not mention | No specification |
| Coffey^S4b^ | 2020 | United States | Jan 1, 2012 - Dec 31, 2016 | T40.0X1, T40.0X4, T40.2X1, T40.2X4, T40.3X1, T40.3X4, T40.4X1, T40.4X4, T40.601, T40.604, T40.691, T40.694, T40.1X1, T40.1X4 | No mention of fatal overdoses | Other set number (e.g., first 5 positions) | A |
| Cole^S180^ | 2021 | United States | Jan 1, 2015 - Dec 31, 2017 | 965.00, 965.01, 965.02, 965.09, E850.0, E850.1, E850.2, E935.0, E935.1, E935.2, T40.0X1, T40.0X2, T40.0X3, T40.0X4, T40.1X1, T40.1X2, T40.1X3, T40.1X4, T40.2X1, T40.2X2, T40.2X3, T40.2X4, T40.3X1, T40.3X2, T40.3X3, T40.3X4, T40.4X1, T40.4X2, T40.4X3, T40.4X4, T40.601, T40.602, T40.603, T40.604, T40.691, T40.692, T40.693, T40.694 | No | Any code field | A |
| Deng^S181^ | 2021 | United States | Jan 1, 2009 - Dec 31, 2017 | 965.0, 965.00, 965.01, 965.02, 965.09, 970.1, E850.0, E850.1, E850.2, E935.0, E935.1, E935.2, E940.1, T40.0X1, T40.0X2, T40.0X3, T40.0X4, T40.0X5, T40.1X1, T40.1X2, T40.1X3, T40.1X4, T40.2X1, T40.2X2, T40.2X3, T40.2X4, T40.2X5, T40.3X1, T40.3X2, T40.3X3, T40.3X4, T40.3X5, T40.4X1, T40.4X2, T40.4X3, T40.4X4, T40.4X5, T40.601, T40.602, T40.603, T40.604, T40.605, T40.691, T40.692, T40.693, T40.694, T40.695 | No mention of fatal overdoses | Did not mention | A; S; D |
| Esposito^S182^ | 2020 | United States | Jul 2010 - Sept 2016 | 965.00, 965.02, 965.09, E850.1, E850.2, T40.0, T40.2, T40.3, T40.4, X42, X62, Y12 | Yes | Any code field | No specification |
| Glanz^S183^ | 2019 | United States | Jan 1, 2006 - Dec 31, 2017 | 965.01, E850.0, 965.0, 965.00, 965.02, 965.09, E850.1, E850.2, T40.1X1, T40.1X2, T40.1X3, T40.1X4, T40.0X1, T40.0X2, T40.0X3, T40.0X4, T40.2X1, T40.2X2, T40.2X3, T40.2X4, T40.3X1, T40.3X2, T40.3X3, T40.3X4, T40.4X1, T40.4X2, T40.4X3, T40.4X4 | Yes | Did not mention | No specification |
| Griffith^S184^ | 2023 | United States | 2021 | 965.00, 965.01, 965.02, 965.09, E850.0, E850.1, E850.2, T40.1X1, T40.1X4, T40.0X1, T40.0X4, T40.2X1, T40.2X4, T40.3X1, T40.3X4, T40.4X1, T40.4X4, T40.411, T40.414, T40.421, T40.424, T40.491, T40.494, T40.601, T40.604, T40.691, T40.694, F11.12, F11.120, F11.121, F11.122, F11.129, F11.22, F11.220, F11.221, F11.222, F11.229, F11.92, F11.920, F11.921, F11.922, F11.929, F11.10, F11.90, F11.20 | No mention of fatal overdoses | Did not mention | A |
| Hartung^S185^ | 2023 | United States | 2014 - 2018 | 965.00, 965.01, 965.02, 965.09, E850.0, E850.1, E850.2, T40.0X, T40.1X, T40.2X, T40.3X, T40.4X | No | Did not mention | No specification |
| Holland^S48^ | 2021 | United States | Dec 30, 2018 - Oct 10, 2020 | 965.00, 965.01, 965.02, 965.09,E850.0, E850.1, E850.2, F11.12, F11.120, F11.121, F11.122, F11.129, F11.22, F11.220, F11.221, F11.222, F11.229, F11.92, F11.920, F11.921, F11.922, F11.929, T40.1X1, T40.1X4, T40.0X1, T40.0X4, T40.2X1, T40.2X4, T40.3X1, T40.3X4, T40.4X1, T40.4X4, T40.601, T40.604, T40.691, T40.694, F11.10, F11.90, F11.20 |  | Any code field | A |
| Hoots^S186^ | 2019 | United States | 2006 - 2016 | 965.00, 965.01, 965.02, 965.09, E850.0, E850.1, E850.2, T40.0X1, T40.0X2, T40.0X3, T40.0X4, T40.1X1, T40.1X2, T40.1X3, T40.1X4, T40.2X1, T40.2X2, T40.2X3, T40.2X4, T40.3X1, T40.3X2, T40.3X3, T40.3X4, T40.4X1, T40.4X2, T40.4X3, T40.4X4, T40.601, T40.602, T40.603, T40.604, T40.691, T40.692, T40.693, T40.694 | No | Any code field | A; S; D |
| Khan^S187^ | 2022 | United States | Jan 1, 2000 - Dec 31, 2019 | 956.00, 965.02, 965.09, E850.1, E850.2, T40.0X1, T40.0X2, T40.0X3, T40.0X4, T40.2X1, T40.2X2, T40.2X3, T40.2X4, T40.3X1, T40.3X2, T40.3X3, T40.3X4, T40.4X1, T40.4X2, T40.4X3, T40.4X4, T40.601, T40.602, T40.603, T40.604, T40.691, T40.692, T40.693, T40.694 | No mention of fatal overdoses | Did not mention | A; S; D |
| Kilaru^S188^ | 2020 | United States | Oct 1, 2011 - Sept 30, 2016 | 965.01, E850.0, 965.00, 965.02, 965.09, E850.1, E850.2, T40.1x, T40.0x, T40.2x, T40.3x, T40.4x, T40.6x | No | Did not mention | No specification |
| Kolak^S189^ | 2020 | United States | 2015 - 2017 | E935.0, E935.1, E935.2, E940.1, 965.00, 965.01, 965.02, 965.09, E850.0, E850.1, E850.2, 970.1, T40.0X1, T40.0X4, T40.0X5, T40.1X1, T40.1X4, T40.2X1, T40.2X4, T40.2X5, T40.3X1, T40.3X4, T40.3X5, T40.4X1, T40.4X4, T40.4X5, T40.601, T40.604, T40.605, T40.691, T40.694, T40.695 | No | Did not mention | A; S; D |
| Krans^S190^ | 2021 | United States | 2009 - 2017 | 965.00, 965.01, 965.02, 965.09, E850.0, E850.1, E850.2, E935.0, E935.1, E935.2, T40.0X1, T40.0X2, T40.0X3, T40.0X4, T40.1X1, T40.1X2, T40.1X3, T40.1X4, T40.2X1, T40.2X2, T40.2X3, T40.2X4, T40.3X1, T40.3X2, T40.3X3, T40.3X4, T40.4X1, T40.4X2, T40.4X3, T40.4X4, T40.601, T40.602, T40.603, T40.604, T40.691, T40.692, T40.693, T40.694 | No mention of fatal overdoses | Did not mention | A |
| Larochelle^S191^ | 2022 | United States | Jan 1, 2010 - Dec 31, 2018 | 965.0x, E850.0, E850.1, E850.2, T40.0X1, T40.0X2, T40.0X4, T40.0X5, T40.1X1, T40.1X2, T40.1X4, T40.2X1, T40.2X2, T40.2X4, T40.2X5, T40.3X1, T40.3X2, T40.3X4, T40.3X5, T40.4X1, T40.4X2, T40.4X4, T40.4X5 | No mention of fatal overdoses | Did not mention | No specification |
| Lo-Ciganic^S192^ | 2022 | United States | 2013-2018 | 965.00, 965.01, 965.02, 965.09, E850.0, E850.1, E850.2, E935.0, E935.1, E935.2, T40.0X1, T40.0X2, T40.0X3, T40.0X4, T40.1X1, T40.1X2, T40.1X3, T40.1X4, T40.2X1, T40.2X2, T40.2X3, T40.2X4, T40.3X1, T40.3X2, T40.3X3, T40.3X4, T40.4X1, T40.4X2, T40.4X3, T40.4X4, T40.601, T40.602, T40.603, T40.604, T40.691, T40.692, T40.693, T40.694 | No | Other | A |
| Lo-Ciganic^S193^ | 2022 | United States | 2013 - 2016 | 965.00, 965.01, 965.02, 965.09, E850.0, E850.1, E850.2, E935.0, E935.1, E935.2, T40.0X1, T40.0X2, T40.0X3, T40.0X4, T40.1X1, T40.1X2, T40.1X3, T40.1X4, T40.2X1, T40.2X2, T40.2X3, T40.2X4, T40.3X1, T40.3X2, T40.3X3, T40.3X4, T40.4X1, T40.4X2, T40.4X3, T40.4X4, T40.601, T40.602, T40.603, T40.604, T40.691, T40.692, T40.693, T40.694 | No mention of fatal overdoses | Did not mention | A |
| Lobo^S194^ | 2020 | United States | 2007 - 2015 | 965.00, 965.01, 965.02, 965.09, E850.0, E850.1, E850.2, T40.0X1, T40.0X3, T40.0X4, T40.1X, T40.1X1, T40.1X3, T40.1X4, T40.2X1, T40.2X3, T40.2X4, T40.2X5, T40.3X1, T40.3X3, T40.3X4, T40.3X5, T40.4X1, T40.4X3, T40.4X4, T40.4X5, T40.601, T40.603, T40.604, T40.691, T40.693, T40.694 | No mention of fatal overdoses | Did not mention | A; S; D |
| Lyden^S195^ | 2023 | United States | Jan 2006 - Jun 2019 | 965.00, 965.01, 965.02, 965.09, E850.0, E850.1, E850.2, T40.0X1, T40.0X2, T40.0X3, T40.0X4, T40.1X1, T40.1X2, T40.1X3, T40.1X4, T40.2X1, T40.2X2, T40.2X3, T40.2X4, T40.3X1, T40.3X2, T40.3X3, T40.3X4, T40.4X1, T40.4X2, T40.4X3, T40.4X4 | Yes | Did not mention | No specification |
| Marino^S196^ | 2019 | United States | 2016 - 2018 | 965.00, 965.01, 965.02, 965.09, E850.0, E850.1, E850.2, T40.0, T40.1, T40.2, T40.3, T40.4, T40.6, T40.69, F11.12, F11.22, F11.92, T40.0X, T40.0X1, T40.0X2, T40.0X3, T40.0X4, T40.0X5, T40.0X6, F11.120, F11.121, F11.122, F11.129, F11.22, F11.220, F11.221, F11.222, F11.229, F11.92, F11.920, F11.921, F11.922, F11.929 | No | Did not mention | A; S; D |
| Morgan^S197^ | 2019 | United States | 2010 - 2016 | 965.0x, E850.0, E850.1, E850.2, T40.0X1, T40.1X1, T40.2X1, T40.3X1, T40.4X1, T40.6X1 | Yes | Did not mention | A; S; D |
| Powell^S198^ | 2023 | United States | 2012 - 2017 | 965.00, 965.01, 965.02, 965.09, 970.1, E850.0, E850.1, E850.2, T40.0X1, T40.0X4, T40.1X1, T40.1X4, T40.2X1, T40.2X4, T40.3X1, T40.3X4, T40.4X1, T40.4X4, T40.601, T40.604, T40.691, T40.694 | No mention of fatal overdoses | Did not mention | A; S; D |
| Qeadan^S199^ | 2024 | United States | 2012 - 2022 | 965.00, 965.01, 965.02, 965.09, E850.0, E850.1, E850.2, 965.0, F11.12, F11.120, F11.121, F11.122, F11.129, F11.22, F11.220, F11.221, F11.222, F11.229, F11.92, F11.920, F11.921, F11.922, F11.929, T40.0X1, T40.0X2, T40.0X3, T40.0X4, T40.1X1, T40.1X2, T40.1X3, T40.1X4, T40.2X1, T40.2X2, T40.2X3, T40.2X4, T40.3X1, T40.3X2, T40.3X3, T40.3X4, T40.411, T40.412, T40.413, T40.414, T40.421, T40.422, T40.423, T40.424, T40.491, T40.492, T40.493, T40.494, T40.4X1, T40.4X2, T40.4X3, T40.4X4, T40.601, T40.602, T40.603, T40.604, T40.691, T40.692, T40.693, T40.694 | No | Did not mention | A; S; D |
| Qeadan^S200^ | 2024 | United States | Jan 2014 - Sept 2022 | 965.00, 965.01, 965.02, 965.09, E850.0, E850.1, E850.2, 965.0, F11.12, F11.120, F11.121, F11.122, F11.129, F11.22, F11.220, F11.221, F11.222, F11.229, F11.92, F11.920, F11.921, F11.922, F11.929, T40.0X1, T40.0X2, T40.0X3, T40.0X4, T40.1X1, T40.1X2, T40.1X3, T40.1X4, T40.2X1, T40.2X2, T40.2X3, T40.2X4, T40.3X1, T40.3X2, T40.3X3, T40.3X4, T40.411, T40.412, T40.413, T40.414, T40.421, T40.422, T40.423, T40.424, T40.491, T40.492, T40.493, T40.494, T40.4X1, T40.4X2, T40.4X3, T40.4X4, T40.601, T40.602, T40.603, T40.604, T40.691, T40.692, T40.693, T40.694 | No mention of fatal overdoses | Did not mention | A; S; D |
| Rebbe^S201^ | 2020 | United States | 2005 - 2017 | 965.00, 965.02, 965.09, 965.01, E850.1, E850.2, E850.0, T40.0X, T40.1X, T40.2X, T40.3X, T40.4X, T40.60, T40.69 | Yes | Did not mention | No specification |
| Ripperger^S202^ | 2022 | United States | 2012 - 2017 | 965.00, 965.01, 965.02, 965.09, E850.0, E850.1, E850.2, T40.0, T40.1, T40.2, T40.3, T40.4, T40.6, T40.0X1, T40.0X2, T40.0X3, T40.0X4, T40.1X1, T40.1X2, T40.1X3, T40.1X4, T40.2X1, T40.2X2, T40.2X3, T40.2X4, T40.3X1, T40.3X2, T40.3X3, T40.3X4, T40.4X1, T40.4X2, T40.4X3, T40.4X4, T40.601, T40.602, T40.603, T40.604, T40.691, T40.692, T40.693, T40.694 | No | Did not mention | A; S; D |
| Samples^S203^ | 2023 | United States | 2008 - 2016 | 965.0, E850.0, E850.1, E850.2, T40.0, T40.1, T40.2, T40.3, T40.4, T40.6 | No mention of fatal overdoses | Did not mention | No specification |
| Schoenfeld^S204^ | 2019 | United States | 2010 - 2016 | 965.0, 965.00, 965.01, 965.02, 965.09, E850.0, E850.1, E850.2, T40.0X1, T40.0X2, T40.0X3, T40.0X4, T40.0X5, T40.1X1, T40.1X2, T40.1X3, T40.1X4, T40.2X1, T40.2X2, T40.2X3, T40.2X4, T40.2X5, T40.3X1, T40.3X2, T40.3X3, T40.3X4, T40.3X5, T40.4X1, T40.4X2, T40.4X3, T40.4X4, T40.4X5 | No mention of fatal overdoses | Any code field | A; S |
| Siglin^S205^ | 2020 | United States | Jan 1, 2007 - Dec 31, 2015 | 965.00, 965.01, 965.02, 965.09, E850.0, E850.1, E850.2, E935.2, T40.0X1, T40.0X2, T40.0X4, T40.1X1, T40.1X2, T40.1X4, T40.2X1, T40.2X2, T40.2X4, T40.3X1, T40.3X2 | No mention of fatal overdoses | Did not mention | A; S; D |
| Smith^S206^ | 2023 | United States | Jan 1, 2014 - Dec 31, 2017 | 965.00, 965.01, 965.02, 965.09, E850.0, E850.1, E850.2, E935.0, E935.1, E935.2, T40.0X1, T40.0X2, T40.0X3, T40.0X4, T40.1X1, T40.1X2, T40.1X3, T40.1X4, T40.2X1, T40.2X2, T40.2X3, T40.2X4, T40.3X1, T40.3X2, T40.3X3, T40.3X4, T40.4X1, T40.4X2, T40.4X3, T40.4X4, T40.601, T40.602, T40.603, T40.604, T40.691, T40.692, T40.693, T40.694 | Yes | Did not mention | A |
| Smith^S207^ | 2024 | United States | Jan 1, 2014 - Dec 31, 2020 | 965.00, 965.01, 965.02, 965.09, E850.0, E850.1, E850.2, E935.0, E935.1, E935.2, T40.0X1, T40.0X2, T40.0X3, T40.0X4, T40.1X1, T40.1X2, T40.1X3, T40.1X4, T40.2X1, T40.2X2, T40.2X3, T40.2X4, T40.3X1, T40.3X2, T40.3X3, T40.3X4, T40.4X1, T40.4X2, T40.4X3, T40.4X4, T40.601, T40.602, T40.603, T40.604, T40.691, T40.692, T40.693, T40.694 | Yes | Did not mention | No specification |
| Tipping^S208^ | 2024 | United States | Jul 2012 - Dec 2021 | 965.01, E850.0, 965.02, E850.1, 965.00, 965.09, E850.2, T40.1, T40.4, T40.3, T40.0, T40.2, T40.6 | No mention of fatal overdoses | Did not mention | No specification |
| Treitler^S209^ | 2024 | United States | Jan 2015 - Jun 2020 | 965.01, E850.0, 965.00, 965.02, 965.09, 970.1, E850.1, E850.2, E935.0, E935.1, E935.2, E940.1, T40.0X1, T40.0X2, T40.0X3, T40.0X4, T40.0X5, T40.1X1, T40.1X2, T40.1X3, T40.1X4, T40.2X1, T40.2X2, T40.2X3, T40.2X4, T40.2X5, T40.3X1, T40.3X2, T40.3X3, T40.3X4, T40.3X5, T40.4X1, T40.4X2, T40.4X3, T40.4X4, T40.4X5, T40.601, T40.602, T40.603, T40.604, T40.605, T40.691, T40.692, T40.693, T40.694, T40.695, T40.411, T40.412, T40.413, T40.414, T40.415, T40.421, T40.422, T40.423, T40.424, T40.425, T40.491, T40.492, T40.493, T40.494, T40.495 | No mention of fatal overdoses | Did not mention | No specification |
| Ward^S210^ | 2023 | United States | Jan 1, 2012 - Apr 30, 2021 | 965.00, 965.01, 965.02, 965.09, E850.0, E850.1, E850.2, E935.0, E935.1, T40.0X1, T40.0X2, T40.0X3, T40.0X4, T40.1X1, T40.1X2, T40.1X3, T40.1X4, T40.1X5, T40.2X1, T40.2X2, T40.2X3, T40.2X4, T40.3X1, T40.3X2, T40.3X3, T40.3X4, T40.3X5 | No mention of fatal overdoses | Did not mention | A; S; D |
| Wei^S211^ | 2019 | United States | 2005 - 2016 | 965.0x, E850.0, E850.1, E850.2, T40.0X1, T40.0X4, T40.1X1, T40.1X4, T40.2X1, T40.2X4, T40.3X1, T40.3X4, T40.4X1, T40.4X4, T40.601, T40.604, T40.691, T40.694 | No mention of fatal overdoses | Any code field | A; S |
| Wei^S212^ | 2019 | United States | 2005 - 2016 | 965.0x, E850.0, E850.1, E850.2, T40.0X1, T40.0X4, T40.1X1, T40.1X4, T40.2X1, T40.2X4, T40.3X1, T40.3X4, T40.4X1, T40.4X4, T40.601, T40.604, T40.691, T40.694 | No mention of fatal overdoses | Any code field | A; S |
| Yang^S213^ | 2021 | United States | Jan 2010 - Oct 2015 | 965.00, 965.01, 965.02, 965.09, E850.0, E850.1, E850.2, T40.0X1, T40.0X2, T40.0X3, T40.0X4, T40.1X1, T40.1X2, T40.1X3, T40.1X4, T40.2X1, T40.2X2, T40.2X3, T40.2X4, T40.3X1, T40.3X2, T40.3X3, T40.3X4, T40.4X1, T40.4X2, T40.4X3, T40.4X4, T40.601, T40.602, T40.603, T40.604, T40.691, T40.692, T40.693, T40.694 | No mention of fatal overdoses | Any code field | A |
| Yunusa^S214^ | 2022 | United States | 2000 - 2020 | 965.0x, E850.1, E850.2, T40.0X1, T40.0X2, T40.0X3, T40.0X4, T40.2X1, T40.2X2, T40.2X3, T40.2X4, T40.3X1, T40.3X2, T40.3X3, T40.3X4, T40.4X1, T40.4X2, T40.4X3, T40.4X4, T40.601, T40.602, T40.603, T40.604, T40.691, T40.692, T40.693, T40.694 | No mention of fatal overdoses | Did not mention | A; S; D |

ICD: International Classification of Diseases; ICD-9-CM: International Classification of Diseases, Ninth Edition, Clinical Modification; ICD-10-CM: International Classification of Diseases, Tenth Edition, Clinical Modification; ICD-10-CA: International Classification of Diseases, Tenth Revision, Canada

^a^Reported ICD-10 in-text but ICD-10-CM in tables; we have interpreted as ICD-10-CM, but it is possible the authors intended to report ICD-10.

^b^Described using ICD-9-CM codes, but not reported in text.

**Supplemental 1.** Reference list for all studies included in this review.

S1. Brown R, Riley MR, Ulrich L, et al. Impact of New York prescription drug monitoring program, I-STOP, on statewide overdose morbidity. *Drug and Alcohol Dependence*. 2017;178:348–354. doi:10.1016/j.drugalcdep.2017.05.023

S2. Vivolo-Kantor AM, Hoots B, David F, Gladden RM. Suspected Heroin Overdoses in US Emergency Departments, 2017-2018. *American journal of public health*. 2019;109(7):1022–1024. doi:10.2105/AJPH.2019.305053

S3. Krishnaswami S, Mukhopadhyay S, McPheeters M, Nechuta SJ. Prescribing patterns before and after a non-fatal drug overdose using Tennessee's controlled substance monitoring database linked to hospital discharge data. *Preventive Medicine*. 2020;130doi:10.1016/j.ypmed.2019.105883

S4. Coffey W, Hunter A, Mobley E, Vivolo-Kantor A. Rural-Urban Trends in Opioid Overdose Discharges in Missouri Emergency Departments, 2012-2016. *J Rural Health*. 2020;36(2):177–186. doi:10.1111/jrh.12368

S5. Lagisetty P, Zhang K, Haffajee RL, et al. Opioid prescribing history prior to heroin overdose among commercially insured adults. *Drug and Alcohol Dependence*. 2020;212doi:10.1016/j.drugalcdep.2020.108061

S6. Mayberry S, Nechuta S, Krishnaswami S. Impact of benzodiazepines and polysubstance status on repeat non-fatal drug overdoses. *Journal of Substance Abuse Treatment*. 2021;123doi:10.1016/j.jsat.2021.108285

S7. Scholl L, Liu S, Vivolo-Kantor A, et al. Development and Validation of a Syndrome Definition to Identify Suspected Nonfatal Heroin-Involved Overdoses Treated in Emergency Departments. *J Public Health Manag Pract*. 2021;27(4):369–378. doi:10.1097/PHH.0000000000001271

S8. Krishnaswami S, Mukhopadhyay S, Markus SA, Nechuta SJ. Prescription Opioid Characteristics and Nonfatal Overdose Among Patients Discharged from Tennessee Emergency Departments. *Journal of Emergency Medicine*. 2022;62(1):51–63. doi:10.1016/j.jemermed.2021.07.050

S9. Hartung DM, Geddes J, Hallvik SE, et al. Evaluation of a Medicaid performance improvement project to reduce high-dose opioid prescriptions. *BMC Health Serv Res*. 2022;22(1)doi:10.1186/s12913-022-07477-6

S10. Chen WW, Page TF, Sun WJ. Racial/Ethnic Disparities in Opioid Use Disorder and Poisoning Emergency Department Visits in Florida. *J Racial Ethn Health Disparities*. 2021;8(6):1395–1405. doi:10.1007/s40615-020-00901-9

S11. Chang MH, Moonesinghe R, Schieber LZ, Truman BI. Opioid-related diagnoses and concurrent claims for HIV, HBV, or HCV among medicare beneficiaries, United States, 2015. *Journal of Clinical Medicine*. 2019;8(11)doi:10.3390/jcm8111768

S12. Casillas SM, Pickens CM, Tanz LJ, Vivolo-Kantor AM. Estimating the ratio of fatal to non-fatal overdoses involving all drugs, all opioids, synthetic opioids, heroin or stimulants, USA, 2010-2020. *Inj Prev*. 2024;30(2):114–124. doi:10.1136/ip-2023-045091

S13. Peterson C, Liu Y, Xu L, Nataraj N, Zhang K, Mikosz CA. U.S. National 90-Day Readmissions After Opioid Overdose Discharge. *American Journal of Preventive Medicine*. 2019;56(6):875–881. doi:10.1016/j.amepre.2018.12.003

S14. Jairam V, Yang DX, Yu JB, Park HS. Emergency department visits for opioid overdoses among patients with cancer. *Journal of the National Cancer Institute*. 2020;112(9):938–943. doi:10.1093/jnci/djz233

S15. Slavova S, Quesinberry D, Costich JF, et al. ICD-10-CM–Based Definitions for Emergency Department Opioid Poisoning Surveillance: Electronic Health Record Case Confirmation Study. *Public Health Reports*. 2020;135(2):262–269. doi:10.1177/0033354920904087

S16. El Ibrahimi S, Hendricks MA, Little K, et al. The association between community social vulnerability and prescription opioid availability with individual opioid overdose. *Drug and Alcohol Dependence*. 2023;252:1–7. doi:10.1016/j.drugalcdep.2023.110991

S17. Peters JL, Durand WM, Monteiro KA, Dumenco L, George P. Opioid Overdose Hospitalizations among Medicare-Disability Beneficiaries. *J Am Board Fam Med*. 2018;31(6):881–896. doi:10.3122/jabfm.2018.06.180152

S18. Sakhuja A, Sztajnkrycer M, Vallabhajosyula S, Cheungpasitporn W, Patch R, Jentzer J. National trends and outcomes of cardiac arrest in opioid overdose. *Resuscitation*. 2017;121:84–89. doi:10.1016/j.resuscitation.2017.10.010

S19. Alinsky RH, Zima BT, Rodean J, et al. Receipt of Addiction Treatment after Opioid Overdose among Medicaid-Enrolled Adolescents and Young Adults. *JAMA Pediatrics*. 2020;174(3)doi:10.1001/jamapediatrics.2019.5183

S20. Guy GP, Zhang K. Effect of State Policy Changes in Florida on Opioid-Related Overdoses. *American Journal of Preventive Medicine*. 2020;58(5):703–706. doi:10.1016/j.amepre.2019.11.008

S21. Mosher H, Zhou Y, Thurman AL, Sarrazin MV, Ohl ME. Trends in Hospitalization for Opioid Overdose among Rural Compared to Urban Residents of the United States, 2007-2014. *J Hosp Med*. 2017;12(11):925–929. doi:10.12788/jhm.2793

S22. Unick GJ, Ciccarone D. US regional and demographic differences in prescription opioid and heroin-related overdose hospitalizations. *International Journal of Drug Policy*. 2017;46:112–119. doi:10.1016/j.drugpo.2017.06.003

S23. Pear VA, Ponicki WR, Gaidus A, et al. Urban-rural variation in the socioeconomic determinants of opioid overdose. *Drug and Alcohol Dependence*. 2019;195:66–73. doi:10.1016/j.drugalcdep.2018.11.024

S24. Hartung DM, Johnston KA, Hallvik S, et al. Prescription Opioid Dispensing Patterns Prior to Heroin Overdose in a State Medicaid Program: a Case-Control Study. *Journal of General Internal Medicine*. 2020;35(11):3188–3196. doi:10.1007/s11606-020-06192-4

S25. Ji X, Haight SC, Ko JY, et al. Association between State Policies on Improving Opioid Prescribing in 2 States and Opioid Overdose Rates among Reproductive-aged Women. *Medical Care*. 2021;59(2):185–192. doi:10.1097/MLR.0000000000001475

S26. Meiman J, Tomasallo C, Paulozzi L. Trends and characteristics of heroin overdoses in Wisconsin, 2003-2012. *Drug Alcohol Depend*. 2015;152:177–84. doi:10.1016/j.drugalcdep.2015.04.002

S27. Guy GP, Jr., Pasalic E, Zhang K. Emergency Department Visits Involving Opioid Overdoses, U.S., 2010-2014. *Am J Prev Med*. 2018;54(1):e37–e39. doi:10.1016/j.amepre.2017.09.003

S28. Unick G, Rosenblum D, Mars S, Ciccarone D. The relationship between US heroin market dynamics and heroin-related overdose, 1992-2008. *Addiction (Abingdon, England)*. 2014;109(11):1889–1898. doi:10.1111/add.12664

S29. Unick GJ, Rosenblum D, Mars S, Ciccarone D. Intertwined Epidemics: National Demographic Trends in Hospitalizations for Heroin- and Opioid-Related Overdoses, 1993-2009. *PLoS ONE*. 2013;8(2)doi:10.1371/journal.pone.0054496

S30. Sears JM, Fulton-Kehoe D, Schulman BA, Hogg-Johnson S, Franklin GM. Opioid Overdose Hospitalization Trajectories in States With and Without Opioid-Dosing Guidelines. *Public Health Reports*. 2019;134(5):567–576. doi:10.1177/0033354919864362

S31. Hsu DJ, McCarthy EP, Stevens JP, Mukamal KJ. Hospitalizations, costs and outcomes associated with heroin and prescription opioid overdoses in the United States 2001-12. *Addiction*. 2017;112(9):1558–1564. doi:10.1111/add.13795

S32. Castillo-Carniglia A, Ponicki WR, Gaidus A, et al. Prescription Drug Monitoring Programs and Opioid Overdoses: Exploring Sources of Heterogeneity. *Epidemiology*. 2019;30(2):212–220. doi:10.1097/EDE.0000000000000950

S33. Mair C, Sumetsky N, Burke JG, Gaidus A. Investigating the Social Ecological Contexts of Opioid Use Disorder and Poisoning Hospitalizations in Pennsylvania. *Journal of studies on alcohol and drugs*. 2018;79(6):899–908. doi:10.15288/jsad.2018.79.899

S34. Pauly NJ, Slavova S, Delcher C, Freeman PR, Talbert J. Features of prescription drug monitoring programs associated with reduced rates of prescription opioid-related poisonings. *Drug and alcohol dependence*. 2018;184(ebs, 7513587):26–32. doi:https://dx.doi.org/10.1016/j.drugalcdep.2017.12.002

S35. Modarai F, Mack K, Hicks P, et al. Relationship of opioid prescription sales and overdoses, North Carolina. *Drug and Alcohol Dependence*. 2013;132(1-2):81–86. doi:10.1016/j.drugalcdep.2013.01.006

S36. Tadros A, Layman SM, Davis SM, Davidov DM, Cimino S. Emergency visits for prescription opioid poisonings. *Journal of Emergency Medicine*. 2015;49(6):871–877. doi:10.1016/j.jemermed.2015.06.035

S37. Burton BN, Lin TC, Said ET, Gabriel RA. National Trends and Factors Associated With Inpatient Mortality in Adult Patients With Opioid Overdose. *Anesthesia and Analgesia*. 2019;128(1):152–160. doi:10.1213/ANE.0000000000003755

S38. Burton BN, Labastide AS, Meineke MN, Schmidt UH, Gabriel RA. Cardiorespiratory morbidity and mortality of opioid overdose during admission to safety-net hospitals. *Journal of Clinical Anesthesia*. 2019;54:66–71. doi:10.1016/j.jclinane.2018.10.036

S39. Nguyen AP, Glanz JM, Narwaney KJ, Binswanger IA. Association of Opioids Prescribed to Family Members with Opioid Overdose among Adolescents and Young Adults. *JAMA Network Open*. 2020;doi:10.1001/jamanetworkopen.2020.1018

S40. Fulton-Kehoe D, Sullivan MD, Turner JA, et al. Opioid Poisonings in Washington State Medicaid: Trends, Dosing, and Guidelines. *Medical Care*. 2015;53(8):679–685. doi:10.1097/MLR.0000000000000384

S41. Chang HY, Ferris L, Eisenberg M, et al. The impact of various risk assessment time frames on the performance of opioid overdose forecasting models. *Medical Care*. 2020;58(11):1013–1021. doi:10.1097/MLR.0000000000001389

S42. Chang HY, Krawczyk N, Schneider KE, et al. A predictive risk model for nonfatal opioid overdose in a statewide population of buprenorphine patients. *Drug and Alcohol Dependence*. 2019;201:127–133. doi:10.1016/j.drugalcdep.2019.04.016

S43. Casillas SM, Scholl L, Mustaquim D, Vivolo-Kantor A. Analysis of trends and usage of ICD-10-CM discharge diagnosis codes for poisonings by fentanyl, tramadol, and other synthetic narcotics in emergency department data. *Addictive Behaviors Reports*. 2022;16doi:10.1016/j.abrep.2022.100464

S44. Averitt AJ, Slovis BH, Tariq AA, Vawdrey DK, Perotte AJ. Characterizing non-heroin opioid overdoses using electronic health records. *JAMIA Open*. 2020;3(1):77–86. doi:10.1093/JAMIAOPEN/OOZ063

S45. Chua KP, Brummett CM, Conti RM, Bohnert A. Association of Opioid Prescribing Patterns with Prescription Opioid Overdose in Adolescents and Young Adults. *JAMA Pediatrics*. 2020;174(2):141–148. doi:10.1001/jamapediatrics.2019.4878

S46. Li Y, Delcher C, Wei YJJ, et al. Risk of Opioid Overdose Associated With Concomitant Use of Opioids and Skeletal Muscle Relaxants: A Population-Based Cohort Study. *Clinical Pharmacology and Therapeutics*. 2020;108(1):81–89. doi:10.1002/cpt.1807

S47. Chen C, Miano TA, Brensinger CM, Leonard CE, Bilker WB, Hennessy S. Risk of Opioid Overdose Associated with Concomitant Use of Methadone and Statins. *Clinical Pharmacology and Therapeutics*. 2024;doi:10.1002/cpt.3479

S48. Holland KM, Jones C, Vivolo-Kantor AM, et al. Trends in US Emergency Department Visits for Mental Health, Overdose, and Violence Outcomes before and during the COVID-19 Pandemic. *JAMA Psychiatry*. 2021;78(4):372–379. doi:10.1001/jamapsychiatry.2020.4402

S49. Bagley SM, Larochelle MR, Xuan Z, et al. Characteristics and Receipt of Medication Treatment Among Young Adults Who Experience a Nonfatal Opioid-Related Overdose. *Annals of Emergency Medicine*. 2020;75(1):29–38. doi:10.1016/j.annemergmed.2019.07.030

S50. Boscarino JA, Kirchner HL, Pitcavage JM, et al. Factors associated with opioid overdose: a 10-year retrospective study of patients in a large integrated health care system. *Subst Abuse Rehabil*. 2016;7:131–141. doi:10.2147/SAR.S108302

S51. Cochran G, Gordon AJ, Lo-Ciganic WH, et al. An Examination of Claims-based Predictors of Overdose from a Large Medicaid Program. *Medical Care*. 2017;55(3):291–298. doi:10.1097/MLR.0000000000000676

S52. Cochran G, Gordon AJ, Gellad WF, et al. Medicaid prior authorization and opioid medication abuse and overdose. *The American journal of managed care*. 2017;23(5):e164–e171.

S53. Coupet E, Werner RM, Polsky D, Karp D, Delgado MK. Impact of the Young Adult Dependent Coverage Expansion on Opioid Overdoses and Deaths: a Quasi-Experimental Study. *Journal of General Internal Medicine*. 2020;35(6):1783–1788. doi:10.1007/s11606-019-05605-3

S54. Fonda JR, Gradus JL, Brogly SB, McGlinchey RE, Milberg WP, Fredman L. Traumatic Brain Injury and Opioid Overdose among Post-9/11 Veterans with Long-Term Opioid Treatment of Chronic Pain. *Journal of Head Trauma Rehabilitation*. 2020;35(3):209–217. doi:10.1097/HTR.0000000000000546

S55. Frazier W, Cochran G, Lo-Ciganic WH, et al. Medication-assisted treatment and opioid use before and after overdose in Pennsylvania Medicaid. *JAMA*. 2017;318(8):750–752. doi:10.1001/jama.2017.7818

S56. Glanz JM, Narwaney KJ, Mueller SR, et al. Prediction Model for Two-Year Risk of Opioid Overdose Among Patients Prescribed Chronic Opioid Therapy. *Journal of General Internal Medicine*. 2018:1–8. doi:10.1007/s11606-017-4288-3

S57. Green CA, Perrin NA, Janoff SL, Campbell CI, Chilcoat HD, Coplan PM. Assessing the accuracy of opioid overdose and poisoning codes in diagnostic information from electronic health records, claims data, and death records. *Pharmacoepidemiology and Drug Safety*. 2017;26(5):509–517. doi:10.1002/pds.4157

S58. Grossman D, Hamman M. Changes in Opioid Overdose Emergency Encounters Associated with Expansion of Wisconsin Medicaid to Childless Adults in Poverty. *Journal of studies on alcohol and drugs*. 2020;81(6):750–759. doi:10.15288/jsad.2020.81.750

S59. Kuo YF, Raji MA, Liaw V, Baillargeon J, Goodwin JS. Opioid Prescriptions in Older Medicare Beneficiaries After the 2014 Federal Rescheduling of Hydrocodone Products. *Journal of the American Geriatrics Society*. 2018;66(5):945–953. doi:10.1111/jgs.15332

S60. Maeng DD, Han JJ, Fitzpatrick MH, Boscarino JA. Patterns of health care utilization and cost before and after opioid overdose: findings from 10-year longitudinal health plan claims data. *Subst Abuse Rehabil*. 2017;8:57–67. doi:10.2147/SAR.S135884

S61. Meara E, Horwitz JR, Powell W, et al. State legal restrictions and prescription-opioid use among disabled adults. *New England Journal of Medicine*. 2016;375(1):44–53. doi:10.1056/NEJMsa1514387

S62. Morgan JR, Wang J, Barocas JA, et al. Opioid overdose and inpatient care for substance use disorder care in Massachusetts. *Journal of Substance Abuse Treatment*. 2020;112:42–48. doi:10.1016/j.jsat.2020.01.017

S63. Mudumbai SC, Lewis ET, Oliva EM, et al. Overdose risk associated with opioid use upon hospital discharge in Veterans Health administration surgical patients. *Pain Medicine (United States)*. 2019;20(5):1020–1031. doi:10.1093/pm/pny150

S64. Olfson M, Crystal S, Wall M, Wang S, Liu SM, Blanco C. Causes of death after nonfatal opioid overdose. *JAMA Psychiatry*. 2018;75(8):820–827. doi:10.1001/jamapsychiatry.2018.1471

S65. Rose AJ, McBain R, Schuler MS, et al. Effect of Age on Opioid Prescribing, Overdose, and Mortality in Massachusetts, 2011 to 2015. *Journal of the American Geriatrics Society*. 2019;67(1):128–132. doi:10.1111/jgs.15659

S66. Shi L, Truong K, Chen L, Basco W. Burden of non-fatal opioid overdose hospitalizations on Medicaid. *Journal of Substance Use*. 2019;24(6):626–629. doi:10.1080/14659891.2019.1640303

S67. Stevens JP, Wall MJ, Novack L, Marshall J, Hsu DJ, Howell MD. The critical care crisis of opioid overdoses in the United States. *Annals of the American Thoracic Society*. 2017;14(12):1803–1809. doi:10.1513/AnnalsATS.201701-022OC

S68. Sun BC, Lupulescu-Mann N, Charlesworth CJ, et al. Does Prescription Opioid Shopping Increase Overdose Rates in Medicaid Beneficiaries? *Annals of Emergency Medicine*. 2018;71(6):679–687.e3. doi:10.1016/j.annemergmed.2017.10.007

S69. Warfield S, Pollini R, Stokes CM, Bossarte R. Opioid-Related Outcomes in West Virginia, 2008-2016. *American journal of public health*. 2019;109(2):303–305. doi:10.2105/AJPH.2018.304845

S70. Yamamoto A, Needleman J, Gelberg L, Kominski G, Shoptaw S, Tsugawa Y. Association between homelessness and opioid overdose and opioid-related hospital admissions/emergency department visits. *Soc Sci Med*. 2019;242:112585. doi:10.1016/j.socscimed.2019.112585

S71. Yarborough BJH, Stumbo SP, Janoff SL, et al. Understanding opioid overdose characteristics involving prescription and illicit opioids: A mixed methods analysis. *Drug and Alcohol Dependence*. 2016;167:49–56. doi:10.1016/j.drugalcdep.2016.07.024

S72. Alexandridis AA, McCort A, Ringwalt CL, et al. A statewide evaluation of seven strategies to reduce opioid overdose in North Carolina. *Injury Prevention*. 2018;24(1):48–54. doi:10.1136/injuryprev-2017-042396

S73. Archibald P, Subramoney K, Beydoun HA, Harris CM. The impact of obesity in patients hospitalized with opioid/opiate overdose. *Substance Abuse*. 2022;43(1):253–259. doi:10.1080/08897077.2021.1941505

S74. Binswanger IA, Narwaney KJ, Gardner EM, Gabella BA, Calcaterra SL, Glanz JM. Development and evaluation of a standardized research definition for opioid overdose outcomes. *Substance abuse*. 2019;40(1):71–79. doi:10.1080/08897077.2018.1546263

S75. Campbell CI, Bahorik AL, VanVeldhuisen P, Weisner C, Rubinstein AL, Thomas Ray G. Use of a prescription opioid registry to examine opioid misuse and overdose in an integrated health system. *Preventive Medicine*. 2018;110:31–37. doi:10.1016/j.ypmed.2018.01.019

S76. Doshi R, Majmundar M, Kansara T, et al. Frequency of Cardiovascular Events and In-hospital Mortality With Opioid Overdose Hospitalizations. *American Journal of Cardiology*. 2019;124(10):1528–1533. doi:10.1016/j.amjcard.2019.07.068

S77. Durrance CP, Austin AE, Runyan CW, et al. Affordable housing through the Low-Income Housing Tax Credit program and opioid overdose emergency department visits. *Journal of Substance Use and Addiction Treatment*. 2024;158doi:10.1016/j.josat.2023.209249

S78. Evans ME, Person M, Reilley B, et al. Trends in Indicators of Injection Drug Use, Indian Health Service, 2010-2014: A Study of Health Care Encounter Data. *Public Health Reports*. 2020;135(4):461–471. doi:10.1177/0033354920937284

S79. Goldman-Mellor S, Olfson M, Lidon-Moyano C, Schoenbaum M. Mortality Following Nonfatal Opioid and Sedative/Hypnotic Drug Overdose. *American Journal of Preventive Medicine*. 2020;59(1):59–67. doi:10.1016/j.amepre.2020.02.012

S80. Green CA, Perrin NA, Hazlehurst B, et al. Identifying and classifying opioid-related overdoses: A validation study. *Pharmacoepidemiology and Drug Safety*. 2019;28(8):1127–1137. doi:10.1002/pds.4772

S81. Hasegawa K, Brown DFM, Tsugawa Y, Camargo Jr CA. Epidemiology of emergency department visits for opioid overdose: A population-based study. *Mayo Clinic Proceedings*. 2014;89(4):462–471. doi:10.1016/j.mayocp.2013.12.008

S82. Hernandez I, He M, Brooks MM, Zhang Y. Exposure-Response Association Between Concurrent Opioid and Benzodiazepine Use and Risk of Opioid-Related Overdose in Medicare Part D Beneficiaries. *JAMA Network Open*. 2018;1(2):e180919. doi:10.1001/jamanetworkopen.2018.0919

S83. Hoffman EM, Watson JC, St Sauver J, Staff NP, Klein CJ. Association of long-term opioid therapy with functional status, adverse outcomes, and mortality among patients with polyneuropathy. *JAMA Neurology*. 2017;74(7):773–779. doi:10.1001/jamaneurol.2017.0486

S84. Hume B, Gabella B, Hathaway J, et al. Assessment of selected overdose poisoning indicators in health care administrative data in 4 states, 2012. *Public Health Reports*. 2017;132(4):488–495. doi:10.1177/0033354917718061

S85. Jasuja GK, Bettano A, Smelson D, et al. Homelessness and Veteran Status in Relation to Nonfatal and Fatal Opioid Overdose in Massachusetts. *Medical Care*. 2021;59(4):S165–S169. doi:10.1097/MLR.0000000000001437

S86. Khan NF, Bateman BT, Landon JE, Gagne JJ. Association of Opioid Overdose with Opioid Prescriptions to Family Members. *JAMA Internal Medicine*. 2019;179(9):1186–1192. doi:10.1001/jamainternmed.2019.1064

S87. Larochelle MR, Liebschutz JM, Zhang F, Ross-Degnan D, Wharam JF. Opioid prescribing after nonfatal overdose and association with repeated overdose: A cohort study. *Annals of Internal Medicine*. 2016;164(1):1–9. doi:10.7326/M15-0038

S88. Larochelle MR, Bernson D, Land T, et al. Medication for opioid use disorder after nonfatal opioid overdose and association with mortality: A cohort study. *Annals of Internal Medicine*. 2018;169(3):137–145. doi:10.7326/M17-3107

S89. Larochelle MR, Bernstein R, Bernson D, et al. Touchpoints – Opportunities to predict and prevent opioid overdose: A cohort study. *Drug and Alcohol Dependence*. 2019;204doi:10.1016/j.drugalcdep.2019.06.039

S90. Li X, Chang CCH, Donohue JM, Krafty RT. A competing risks regression model for the association between time-varying opioid exposure and risk of overdose. *Statistical Methods in Medical Research*. 2022;31(6):1013–1030. doi:10.1177/09622802221075933

S91. Mountcastle SB, Joyce AR, Sasinowski M, Costello N, Doshi S, Zedler BK. Validation of an administrative claims coding algorithm for serious opioid overdose: A medical chart review. *Pharmacoepidemiology and Drug Safety*. 2019;28(10):1422–1428. doi:10.1002/pds.4886

S92. Nam YH, Bilker WB, DeMayo FJ, Neuman MD, Hennessy S. Incidence rates of and risk factors for opioid overdose in new users of prescription opioids among US Medicaid enrollees: A cohort study. *Pharmacoepidemiology and Drug Safety*. 2020;29(8):931–938. doi:10.1002/pds.5067

S93. Nielsen T, Bernson D, Terplan M, et al. Maternal and infant characteristics associated with maternal opioid overdose in the year following delivery. *Addiction*. 2020;115(2):291–301. doi:10.1111/add.14825

S94. Park TW, Larochelle MR, Saitz R, Wang N, Bernson D, Walley AY. Associations between prescribed benzodiazepines, overdose death and buprenorphine discontinuation among people receiving buprenorphine. *Addiction*. 2020;115(5):924–932. doi:10.1111/add.14886

S95. Reardon JM, Harmon KJ, Schult GC, Staton CA, Waller AE. Use of diagnosis codes for detection of clinically significant opioid poisoning in the emergency department: A retrospective analysis of a surveillance case definition. *BMC Emergency Medicine*. 2016;16(1)doi:10.1186/s12873-016-0075-4

S96. Rowe C, Vittinghoff E, Santos GM, Behar E, Turner C, Coffin PO. Performance Measures of Diagnostic Codes for Detecting Opioid Overdose in the Emergency Department. *Academic Emergency Medicine*. 2017;24(4):475–483. doi:10.1111/acem.13121

S97. Sun JW, Franklin JM, Rough K, et al. Predicting overdose among individuals prescribed opioids using routinely collected healthcare utilization data. *PLoS ONE*. 2020;15(10 October)doi:10.1371/journal.pone.0241083

S98. Weiner SG, Baker O, Bernson D, Schuur JD. One-Year Mortality of Patients After Emergency Department Treatment for Nonfatal Opioid Overdose. *Annals of Emergency Medicine*. 2020;75(1):13–17. doi:10.1016/j.annemergmed.2019.04.020

S99. Young JC, Lund JL, Dasgupta N, Jonsson Funk M. Opioid tolerance and clinically recognized opioid poisoning among patients prescribed extended-release long-acting opioids. *Pharmacoepidemiology and Drug Safety*. 2019;28(1):39–47. doi:10.1002/pds.4572

S100. Bozinoff N, Men S, Kurdyak P, Selby P, Gomes T. Prescribing Characteristics Associated with Opioid Overdose Following Buprenorphine Taper. *JAMA Network Open*. 2022;5(9):E2234168. doi:10.1001/jamanetworkopen.2022.34168

S101. Coupet E, D'Onofrio G, Chawarski M, et al. Emergency department patients with untreated opioid use disorder: A comparison of those seeking versus not seeking referral to substance use treatment. *Drug and Alcohol Dependence*. 2021;219doi:10.1016/j.drugalcdep.2020.108428

S102. Forbes LA, Canner JK, Milio L, Halscott T, Vaught AJ. Association of Patient Sex and Pregnancy Status With Naloxone Administration During Emergency Department Visits. *Obstetrics and Gynecology*. 2021;137(5):855–863. doi:10.1097/AOG.0000000000004357

S103. Gomes T, Khuu W, Craiovan D, et al. Comparing the contribution of prescribed opioids to opioid-related hospitalizations across Canada: A multi-jurisdictional cross-sectional study. *Drug Alcohol Depend*. 2018;191:86–90. doi:10.1016/j.drugalcdep.2018.06.028

S104. Gomes T, Campbell T, Tadrous M, Mamdani MM, Paterson JM, Juurlink DN. Initial opioid prescription patterns and the risk of ongoing use and adverse outcomes. *Pharmacoepidemiology and Drug Safety*. 2021;30(3):379–389. doi:10.1002/pds.5180

S105. Houser C, Huynh D, Jasarevic A, Do MT, Young M, Villeneuve PJ. Do statutory holidays impact the number of opioid-related hospitalizations among Canadian adults? Findings from a national case-crossover study. *Canadian journal of public health = Revue canadienne de sante publique*. 2021;112(1):38–48. doi:10.17269/s41997-020-00384-5

S106. Lebin JA, Huynh L, Morse SC, Jablonowksi K, Hall J, Whiteside LK. Predictors of receiving an emergency department naloxone prescription following an opioid overdose. *Am J Emerg Med*. 2021;46:763–764. doi:10.1016/j.ajem.2020.09.027

S107. Moe J, Chong M, Zhao B, Scheuermeyer FX, Purssell R, Slaunwhite A. Death after emergency department visits for opioid overdose in British Columbia: a retrospective cohort analysis. *CMAJ open*. 2021;9(1):E242–E251. doi:10.9778/cmajo.20200169

S108. Morrow RL, Bassett K, Maclure M, Dormuth CR. Outcomes associated with hospital admissions for accidental opioid overdose in British Columbia: A retrospective cohort study. *BMJ Open*. 2019;9(5)doi:10.1136/bmjopen-2018-025567

S109. Nguyen HV, Mital S, Bugden S, McGinty EE. British Columbia’s Safer Opioid Supply Policy and Opioid Outcomes. *JAMA Internal Medicine*. 2024;184(3):256–264. doi:10.1001/jamainternmed.2023.7570

S110. O'Brien DC, Dabbs D, Dong K, Veugelers PJ, Hyshka E. Patient characteristics associated with being offered take home naloxone in a busy, urban emergency department: a retrospective chart review. *BMC health services research*. 2019;19(1):632. doi:10.1186/s12913-019-4469-3

S111. Patel I, Walter LA, Li L. Opioid overdose crises during the COVID-19 pandemic: implication of health disparities. *Harm Reduction Journal*. 2021;18(1)doi:10.1186/s12954-021-00534-z

S112. Rosenbaum J, Lucas N, Zandrow G, et al. Impact of a shelter-in-place order during the COVID-19 pandemic on the incidence of opioid overdoses. *American Journal of Emergency Medicine*. 2021;41:51–54. doi:10.1016/j.ajem.2020.12.047

S113. Sacco DI, Martins SS. Characteristics of Nonfatal Opioid Overdose in Emergency Department Patients Surviving to Discharge or Hospital Admission. *Journal of studies on alcohol and drugs*. 2023;84(2):230–234. doi:10.15288/jsad.22-00204

S114. Thao V, Helfinstine DA, Sangaralingham LR, Yonekawa Y, Starr MR. Hospitalization, Overdose, and Mortality After Opioid Prescriptions Tied to Ophthalmic Surgery. *Ophthalmology*. 2024;131(8):943–949. doi:10.1016/j.ophtha.2024.01.028

S115. Weiner SG, Little K, Yoo J, et al. Opioid Overdose After Medication for Opioid Use Disorder Initiation Following Hospitalization or ED Visit. *JAMA network open*. 2024;7(7):e2423954. doi:10.1001/jamanetworkopen.2024.23954

S116. Welk B, McClure JA, Clarke C, Vogt K, Campbell J. An Opioid Prescription for Men Undergoing Minor Urologic Surgery Is Associated with an Increased Risk of New Persistent Opioid Use. *European Urology*. 2020;77(1):68–75. doi:10.1016/j.eururo.2019.08.031

S117. Abasilim C, Friedman LS, Karch L, Holloway-Beth A. Trends in non-fatal and fatal opioid overdoses during the first two years of the coronavirus disease–2019 pandemic. *Annals of Epidemiology*. 2024;90:35–41. doi:10.1016/j.annepidem.2023.10.007

S118. Boyer TL, Blosnich JR, Hubbard CC, Sharp LK, Cashy JP, Suda KJ. Comparing Outpatient Opioids, High-Risk Prescribing, and Opioid Poisoning Between Transgender and Cisgender Veterans: A Cross-sectional Analysis. *American Journal of Preventive Medicine*. 2022;63(2):168–177. doi:10.1016/j.amepre.2022.02.011

S119. Brathwaite DM, Wolff CS, Ising AI, Proescholdbell SK, Waller AE. A Mixed-Methods Comparison of a National and State Opioid Overdose Surveillance Definition. *Public Health Reports*. 2021;136(1_suppl):31S–39S. doi:10.1177/00333549211018181

S120. Chen Q, Gopaldas M, Castillo F, et al. Prevalence of Opioid Use Disorder and Opioid Overdose Rates Among People With Mental Illness. *Psychiatr Serv*. 2024;75(10):953–960. doi:10.1176/appi.ps.20230338

S121. Chua KP, Dahlem CHY, Nguyen TD, et al. Naloxone and Buprenorphine Prescribing Following US Emergency Department Visits for Suspected Opioid Overdose: August 2019 to April 2021. *Annals of Emergency Medicine*. 2022;79(3):225–236. doi:10.1016/j.annemergmed.2021.10.005

S122. Chua KP, Brummett CM, Ng S, Bohnert ASB. Association between Receipt of Overlapping Opioid and Benzodiazepine Prescriptions from Multiple Prescribers and Overdose Risk. *JAMA Network Open*. 2021;doi:10.1001/jamanetworkopen.2021.20353

S123. Faude S, Delgado MK, Perrone J, et al. Variability in opioid use disorder clinical presentations and treatment in the emergency department: A mixed-methods study. *American Journal of Emergency Medicine*. 2023;66:53–60. doi:10.1016/j.ajem.2023.01.009

S124. Fix J, Ising AI, Proescholdbell SK, et al. Linking Emergency Medical Services and Emergency Department Data to Improve Overdose Surveillance in North Carolina. *Public Health Reports*. 2021;136(1_suppl):54S–61S. doi:10.1177/00333549211012400

S125. Friedman LS, Abasilim C, Karch L, Jasmin W, Holloway-Beth A. Disparities in fatal and non-fatal opioid-involved overdoses among middle-aged non-Hispanic Black Men and Women. *J Racial Ethn Health Disparities*. 2023;doi:10.1007/s40615-023-01877-y

S126. Harder VS, Plante TB, Koh I, et al. Influence of Opioid Prescription Policy on Overdoses and Related Adverse Effects in a Primary Care Population. *Journal of General Internal Medicine*. 2021;36(7):2013–2020. doi:10.1007/s11606-021-06831-4

S127. Harder VS, Varni SE, Murray KA, et al. Prescription opioid policies and associations with opioid overdose and related adverse effects. *International Journal of Drug Policy*. 2021;97doi:10.1016/j.drugpo.2021.103306

S128. Jones CM, Shoff C, Blanco C, Losby JL, Ling SM, Compton WM. Overdose, Behavioral Health Services, and Medications for Opioid Use Disorder after a Nonfatal Overdose. *JAMA Internal Medicine*. 2024;184(8):954–962. doi:10.1001/jamainternmed.2024.1733

S129. Liu S, Scholl L, Hoots B, Seth P. Nonfatal Drug and Polydrug Overdoses Treated in Emergency Departments - 29 States, 2018-2019. *MMWR Morbidity and mortality weekly report*. 2020;69(34):1149–1155. doi:10.15585/mmwr.mm6934a1

S130. Lloyd J, Treitler P, Lister JJ, Nowels M, Crystal S. Methadone treatment utilization and overdose trends among Medicaid beneficiaries in New Jersey before and during the COVID-19 pandemic. *Journal of Substance Use and Addiction Treatment*. 2024;167doi:10.1016/j.josat.2024.209476

S131. Mannes ZL, Wheeler-Martin K, Terlizzi K, et al. Risks of opioid overdose among New York State Medicaid recipients with chronic pain before and during the COVID-19 pandemic. *Preventive Medicine*. 2023;177:1–7. doi:10.1016/j.ypmed.2023.107789

S132. Martin O, Bird H, Nechuta S. Characteristics associated with adult non-fatal opioid and stimulant overdose and substance use disorder emergency department visits in Michigan. *Drug and Alcohol Dependence Reports*. 2024;13doi:10.1016/j.dadr.2024.100290

S133. Moreland B, Lee R. Emergency Department Visits and Hospitalizations for Selected Nonfatal Injuries Among Adults Aged ≥65 Years - United States, 2018. *MMWR Morbidity and mortality weekly report*. 2021;70(18):661–666. doi:10.15585/mmwr.mm7018a1

S134. Rock PJ, Quesinberry D, Singleton MD, Slavova S. Emergency Medical Services and Syndromic Surveillance: A Comparison With Traditional Surveillance and Effects on Timeliness. *Public Health Reports*. 2021;136(1_suppl):72S–79S. doi:10.1177/00333549211018673

S135. Roth KB, Goplerud DK, Babjak JS, Nguyen JL, Gilbert LR. Examining the association of rurality with opioid-related morbidity and mortality in Georgia: A geospatial analysis. *Journal of Substance Use and Addiction Treatment*. 2024;162doi:10.1016/j.josat.2024.209336

S136. Soares WE, Melnick ER, Nath B, et al. Emergency Department Visits for Nonfatal Opioid Overdose During the COVID-19 Pandemic Across Six US Health Care Systems. *Annals of Emergency Medicine*. 2022;79(2):158–167. doi:10.1016/j.annemergmed.2021.03.013

S137. Sorensen IS, Susi A, Andreason P, et al. Opioid-Related Trends in Active Duty Service Members During the Coronavirus Disease 2019 Pandemic. *Military medicine*. 2023;188(6):567–574. doi:10.1093/milmed/usad245

S138. Spencer MR, White DG, Jackson G. National Hospital Care Survey Demonstration Projects:Mortality Following Nonfatal Opioid Overdose Visits to the Emergency Department. *National health statistics reports*. 2023;(187):1–8.

S139. Stevens MA, Melnick ER, Savitz ST, Jeffery MM, Nath B, Janke AT. National trends in emergency conditions through the Omicron COVID-19 wave in commercial and Medicare Advantage enrollees. *JACEP Open*. 2023;4(4)doi:10.1002/emp2.13023

S140. Vivolo-Kantor AM, Seth P, Gladden RM, et al. Vital Signs: Trends in Emergency Department Visits for Suspected Opioid Overdoses - United States, July 2016-September 2017. *MMWR Morbidity and mortality weekly report*. 2018;67(9):279–285. doi:10.15585/mmwr.mm6709e1

S141. Vivolo-Kantor AM, Hoots BE, Scholl L, et al. Nonfatal Drug Overdoses Treated in Emergency Departments - United States, 2016-2017. *MMWR Morbidity and mortality weekly report*. 2020;69(13):371–376. doi:10.15585/mmwr.mm6913a3

S142. Weiner SG, Price CN, Atalay AJ, et al. A Health System–Wide Initiative to Decrease Opioid-Related Morbidity and Mortality. *Joint Commission Journal on Quality and Patient Safety*. 2019;45(1):3–13. doi:10.1016/j.jcjq.2018.07.003

S143. Weiner SG, Carroll AD, Brisbon NM, et al. Evaluating disparities in prescribing of naloxone after emergency department treatment of opioid overdose. *Journal of Substance Abuse Treatment*. 2022;139doi:10.1016/j.jsat.2022.108785

S144. White DG, Adams NB, Brown AM, et al. Enhancing Identification of Opioid-involved Health Outcomes Using National Hospital Care Survey Data. *Vital and health statistics Ser 1, Programs and collection procedures*. 2021;(188):1–31.

S145. Carnide N, Feng G, Song C, Demers PA, MacLeod JS, Sritharan J. Occupational patterns of opioid-related harms comparing a cohort of formerly injured workers to the general population in Ontario, Canada. *Canadian journal of public health = Revue canadienne de sante publique*. 2024;doi:10.17269/s41997-024-00882-w

S146. Leece P, Chen C, Manson H, et al. One-Year Mortality After Emergency Department Visit for Nonfatal Opioid Poisoning: A Population-Based Analysis. *Ann Emerg Med*. 2020;75(1):20–28. doi:10.1016/j.annemergmed.2019.07.021

S147. O'Connor S, Grywacheski V, Louie K. At-a-glance - Hospitalizations and emergency department visits due to opioid poisoning in Canada. *Health Promot Chronic Dis Prev Can*. 2018;38(6):244–247. doi:10.24095/hpcdp.38.6.04

S148. Rudoler D, Kurdyak P, Gomes T, et al. Evaluating the population-level effects of oxycodone restrictions on prescription opioid utilization in Ontario. *Pharmacoepidemiol Drug Saf*. 2022;31(7):769–778. doi:10.1002/pds.5442

S149. Alobaidi A, Pickard AS, Jarrett JB, Lee TA. Hospitalizations for opioid-related overdose and timing of concurrent opioid and benzodiazepine use: A nested case-control study. *Pharmacotherapy*. 2021;41(9):722–732. doi:10.1002/phar.2608

S150. Andersen MS, Lorenz V, Pant A, Bray JW, Caleb Alexander G. Association of Opioid Utilization Management With Prescribing and Overdose. *American Journal of Managed Care*. 2022;28(2):E63–E68. doi:10.37765/ajmc.2022.88829

S151. Badger J, LaRose E, Mayer J, Bashiri F, Page D, Peissig P. Machine learning for phenotyping opioid overdose events. *Journal of Biomedical Informatics*. 2019;94doi:10.1016/j.jbi.2019.103185

S152. Chang HH, Zhang HS, Latimore AD, et al. Associations between short-term ambient temperature exposure and emergency department visits for amphetamine, cocaine, and opioid use in California from 2005 to 2019. *Environment International*. 2023;181doi:10.1016/j.envint.2023.108233

S153. Cicero TJ, Mendoza M, Cattaneo M, et al. Real-world misuse, abuse, and dependence of abuse-deterrent versus non-abuse-deterrent extended-release morphine in Medicaid non-cancer patients. *Postgraduate Medicine*. 2019;131(3):225–229. doi:10.1080/00325481.2019.1585688

S154. Daoust R, Paquet J, Moore L, et al. Opioid Poisoning and Opioid Use Disorder in Older Trauma Patients. *Clin Interv Aging*. 2020;15:763–770. doi:10.2147/CIA.S252849

S155. Gibbons JB, McCullough JS, Zivin K, Brown ZY, Norton EC. Association Between Buprenorphine Treatment Gaps, Opioid Overdose, and Health Care Spending in US Medicare Beneficiaries With Opioid Use Disorder. *JAMA Psychiatry*. 2022;79(12):1173–1179. doi:10.1001/jamapsychiatry.2022.3118

S156. Hendricks MA, El Ibrahimi S, Ritter GA, et al. Association of Household Opioid Availability with Opioid Overdose. *JAMA Network Open*. 2023;6(3):E233385. doi:10.1001/jamanetworkopen.2023.3385

S157. Lindner SR, Hart K, Manibusan B, McCarty D, McConnell KJ. State- and County-Level Geographic Variation in Opioid Use Disorder, Medication Treatment, and Opioid-Related Overdose Among Medicaid Enrollees. *JAMA health forum*. 2023;4(6):e231574. doi:10.1001/jamahealthforum.2023.1574

S158. Nguyen AP, Glanz JM, Narwaney KJ, et al. Update of a Multivariable Opioid Overdose Risk Prediction Model to Enhance Clinical Care for Long-term Opioid Therapy Patients. *Journal of General Internal Medicine*. 2023;38(12):2678–2685. doi:10.1007/s11606-023-08149-9

S159. Qeadan F, Madden EF. Associations between naloxone prescribing and opioid overdose among patients with acute and chronic pain conditions. *Addiction (Abingdon, England)*. 2022;117(2):457–471. doi:10.1111/add.15643

S160. Saloner B, Chang HY, Krawczyk N, et al. Predictive Modeling of Opioid Overdose Using Linked Statewide Medical and Criminal Justice Data. *JAMA Psychiatry*. 2020;77(11):1155–1162. doi:10.1001/jamapsychiatry.2020.1689

S161. Smolina K, Crabtree A, Chong M, et al. Patterns and history of prescription drug use among opioid-related drug overdose cases in British Columbia, Canada, 2015–2016. *Drug and Alcohol Dependence*. 2019;194:151–158. doi:10.1016/j.drugalcdep.2018.09.019

S162. Suffoletto B, Zeigler A. Risk and protective factors for repeated overdose after opioid overdose survival. *Drug and Alcohol Dependence*. 2020;209doi:10.1016/j.drugalcdep.2020.107890

S163. Sugarman OK, Saloner B, Richards TM, et al. Association of buprenorphine retention and subsequent adverse outcomes following non-fatal overdose: An analysis using statewide linked Maryland databases. *Drug and Alcohol Dependence*. 2024;258doi:10.1016/j.drugalcdep.2024.111281

S164. Sun T, Aroke H, Kogut S, Katenka N, Bratberg J, Buchanan A. Early buprenorphine-naloxone initiation for opioid use disorder reduces opioid overdose, emergency room visits, and healthcare cost compared to late initiation. *American Journal of Drug and Alcohol Abuse*. 2022;48(2):217–225. doi:10.1080/00952990.2021.1981358

S165. Weiner SG, El Ibrahimi S, Hendricks MA, et al. Factors Associated with Opioid Overdose after an Initial Opioid Prescription. *JAMA Network Open*. 2022;5(1):E2145691. doi:10.1001/jamanetworkopen.2021.45691

S166. Weiner SG, Hendricks MA, Ibrahimi SE, et al. Opioid-related overdose and chronic use following an initial prescription of hydrocodone versus oxycodone. *PLoS ONE*. 2022;17(4 April)doi:10.1371/journal.pone.0266561

S167. Williams EC, Frost MC, Bounthavong M, et al. Implementation of Opioid Safety Efforts: Influence of Academic Detailing on Adverse Outcomes Among Patients in the Veterans Health Administration. *Subst Use Addctn J*. 2024;45(4):610–623. doi:10.1177/29767342241243309

S168. Williams AR, Samples H, Crystal S, Olfson M. Acute Care, Prescription Opioid Use, and Overdose Following Discontinuation of Long-Term Buprenorphine Treatment for Opioid Use Disorder. *Am J Psychiatry*. 2020;177(2):117–124. doi:10.1176/appi.ajp.2019.19060612

S169. Agnoli A, Xing G, Tancredi DJ, Magnan E, Jerant A, Fenton JJ. Association of Dose Tapering with Overdose or Mental Health Crisis among Patients Prescribed Long-term Opioids. *JAMA - Journal of the American Medical Association*. 2021;326(5):411–419. doi:10.1001/jama.2021.11013

S170. Arakkal AT, Polgreen LA, Chapman CG, et al. Association between household opioid prescriptions and risk for overdose among family members not prescribed opioids. *Pharmacotherapy*. 2024;44(2):110–121. doi:10.1002/phar.2891

S171. Banta-Green CJ, Coffin PO, Merrill JO, et al. Impacts of an opioid overdose prevention intervention delivered subsequent to acute care. *Inj Prev*. 2019;25(3):191–198. doi:10.1136/injuryprev-2017-042676

S172. Beachler DC, Hall K, Garg R, et al. An evaluation of the effect of the oxycontin reformulation on unintentional fatal and nonfatal overdose. *The Clinical Journal of Pain*. 2022;38(6):396–404. doi:10.1097/AJP.0000000000001034

S173. Bovell-Ammon BJ, Yan S, Dunn D, et al. Prison Buprenorphine Implementation and Postrelease Opioid Use Disorder Outcomes. *JAMA Network Open*. 2024;7(3):E242732. doi:10.1001/jamanetworkopen.2024.2732

S174. Bykov K, Bateman BT, Franklin JM, Vine SM, Patorno E. Association of Gabapentinoids with the Risk of Opioid-Related Adverse Events in Surgical Patients in the United States. *JAMA Network Open*. 2020;doi:10.1001/jamanetworkopen.2020.31647

S175. Chang CY, Jones BL, Hincapie-Castillo JM, et al. Association between trajectories of prescription opioid use and risk of opioid use disorder and overdose among US nonmetastatic breast cancer survivors. *Breast Cancer Research and Treatment*. 2024;204(3):561–577. doi:10.1007/s10549-023-07205-6

S176. Chen X, Hou W, Rashidian S, et al. A large-scale retrospective study of opioid poisoning in New York State with implications for targeted interventions. *Scientific reports*. 2021;11(1):5152. doi:10.1038/s41598-021-84148-2

S177. Cherian T, Lim S, Katyal M, et al. Impact of jail-based methadone or buprenorphine treatment on non-fatal opioid overdose after incarceration. *Drug and Alcohol Dependence*. 2024;259doi:10.1016/j.drugalcdep.2024.111274

S178. Chiuve SE, Kilpatrick RD, Hornstein MD, et al. Chronic opioid use and complication risks in women with endometriosis: A cohort study in US administrative claims. *Pharmacoepidemiology and Drug Safety*. 2021;30(6):787–796. doi:10.1002/pds.5209

S179. Chua KP, Kenney BC, Waljee JF, Brummett CM, Nalliah RP. Dental Opioid Prescriptions and Overdose Risk in Patients and Their Families. *American Journal of Preventive Medicine*. 2021;61(2):165–173. doi:10.1016/j.amepre.2021.02.008

S180. Cole ES, Drake C, DiDomenico E, et al. Patterns of clinic switching and continuity of medication for opioid use disorder in a Medicaid-enrolled population. *Drug and Alcohol Dependence*. 2021;221doi:10.1016/j.drugalcdep.2021.108633

S181. Deng J, Hou W, Dong X, et al. A Large-Scale Observational Study on the Temporal Trends and Risk Factors of Opioid Overdose: Real-World Evidence for Better Opioids. *Drugs Real World Outcomes*. 2021;8(3):393–406. doi:10.1007/s40801-021-00253-8

S182. Esposito DB, Cepeda MS, Holick CN, et al. Incidence of opioid overdose among patients using ER/LA opioid analgesics before and after implementation of the class-wide opioid risk evaluation and mitigation strategy. *Journal of Pain Research*. 2020;13:157–169. doi:10.2147/JPR.S219324

S183. Glanz JM, Binswanger IA, Shetterly SM, Narwaney KJ, Xu S. Association between Opioid Dose Variability and Opioid Overdose among Adults Prescribed Long-term Opioid Therapy. *JAMA Network Open*. 2019;2(4)doi:10.1001/jamanetworkopen.2019.2613

S184. Griffith J, Chambers LC, Hallowell BD, et al. Examination of the Accuracy of Existing Overdose Surveillance Systems. *JAMA Network Open*. 2023;6(6):E2320789. doi:10.1001/jamanetworkopen.2023.20789

S185. Hartung DM, McCracken CM, Nguyen T, Kempany K, Waddell EN. Fatal and nonfatal opioid overdose risk following release from prison: A retrospective cohort study using linked administrative data. *Journal of Substance Use and Addiction Treatment*. 2023;147doi:10.1016/j.josat.2023.208971

S186. Hoots B, Vivolo-Kantor A, Seth P. The rise in non-fatal and fatal overdoses involving stimulants with and without opioids in the United States. *Addiction*. 2020;115(5):946–958. doi:10.1111/add.14878

S187. Khan NF, Bykov K, Barnett ML, Glynn RJ, Vine SM, Gagne JJ. Comparative Risk of Opioid Overdose With Concomitant Use of Prescription Opioids and Skeletal Muscle Relaxants. *Neurology*. 2022;99(13):E1432–E1442. doi:10.1212/WNL.0000000000200904

S188. Kilaru AS, Xiong A, Lowenstein M, et al. Incidence of Treatment for Opioid Use Disorder following Nonfatal Overdose in Commercially Insured Patients. *JAMA Network Open*. 2020;3(5)doi:10.1001/jamanetworkopen.2020.5852

S189. Kolak MA, Chen YT, Joyce S, et al. Rural risk environments, opioid-related overdose, and infectious diseases: A multidimensional, spatial perspective. *International Journal of Drug Policy*. 2020;85doi:10.1016/j.drugpo.2020.102727

S190. Krans EE, Kim JY, Chen Q, et al. Outcomes associated with the use of medications for opioid use disorder during pregnancy. *Addiction*. 2021;116(12):3504–3514. doi:10.1111/add.15582

S191. Larochelle MR, Lodi S, Yan S, Clothier BA, Goldsmith ES, Bohnert ASB. Comparative Effectiveness of Opioid Tapering or Abrupt Discontinuation vs No Dosage Change for Opioid Overdose or Suicide for Patients Receiving Stable Long-term Opioid Therapy. *JAMA Network Open*. 2022;5(8):E2226523. doi:10.1001/jamanetworkopen.2022.26523

S192. Lo-Ciganic WH, Donohue JM, Yang Q, et al. Developing and validating a machine-learning algorithm to predict opioid overdose in Medicaid beneficiaries in two US states: a prognostic modelling study. *The Lancet Digital Health*. 2022;4(6):e455–e465. doi:10.1016/S2589-7500(22)00062-0

S193. Lo-Ciganic WH, Hincapie-Castillo J, Wang T, et al. Dosing profiles of concurrent opioid and benzodiazepine use associated with overdose risk among US Medicare beneficiaries: group-based multi-trajectory models. *Addiction*. 2022;117(7):1982–1997. doi:10.1111/add.15857

S194. Lobo CP, Cochran G, Chang CCH, et al. Associations between the Specialty of Opioid Prescribers and Opioid Addiction, Misuse, and Overdose Outcomes. *Pain Medicine (United States)*. 2020;21(9):1871–1890. doi:10.1093/PM/PNZ234

S195. Lyden JR, Xu S, Narwaney KJ, Glanz JM, Binswanger IA. Opioid Overdose Risk Following Hospital Discharge Among Individuals Prescribed Long-Term Opioid Therapy: a Risk Interval Analysis. *Journal of General Internal Medicine*. 2023;38(11):2560–2567. doi:10.1007/s11606-022-08014-1

S196. Marino R, Landau A, Lynch M, Callaway C, Suffoletto B. Do electronic health record prompts increase take-home naloxone administration for emergency department patients after an opioid overdose? *Addiction*. 2019;114(9):1575–1581. doi:10.1111/add.14635

S197. Morgan JR, Schackman BR, Weinstein ZM, Walley AY, Linas BP. Overdose following initiation of naltrexone and buprenorphine medication treatment for opioid use disorder in a United States commercially insured cohort. *Drug and Alcohol Dependence*. 2019;200:34–39. doi:10.1016/j.drugalcdep.2019.02.031

S198. Powell VD, Macleod C, Sussman J, Lin LA, Bohnert ASB, Lagisetty P. Variation in Clinical Characteristics and Longitudinal Outcomes in Individuals with Opioid Use Disorder Diagnosis Codes. *Journal of General Internal Medicine*. 2023;38(3):699–706. doi:10.1007/s11606-022-07732-w

S199. Qeadan F, Madden EF, English K, et al. Quantifying the Burden of Opioid Use Disorder and Non-fatal Opioid Overdose in American Indian and Alaskan Native Populations Using the Cerner Real-World Data™ Database. *Journal of racial and ethnic health disparities*. 2024;doi:10.1007/s40615-024-02084-z

S200. Qeadan F, McCunn A, Tingey B. The association between glucose-dependent insulinotropic polypeptide and/or glucagon-like peptide-1 receptor agonist prescriptions and substance-related outcomes in patients with opioid and alcohol use disorders: A real-world data analysis. *Addiction*. 2024;doi:10.1111/add.16679

S201. Rebbe R, Bishop AS, Ahn J, Mienko JA. Opioid overdose events and child maltreatment indicators: Differential county-level associations. *Children and Youth Services Review*. 2020;119doi:10.1016/j.childyouth.2020.105671

S202. Ripperger M, Lotspeich SC, Wilimitis D, et al. Ensemble learning to predict opioid-related overdose using statewide prescription drug monitoring program and hospital discharge data in the state of Tennessee. *Journal of the American Medical Informatics Association*. 2022;29(1):22–32. doi:10.1093/jamia/ocab218

S203. Samples H, Nowels MA, Williams AR, Olfson M, Crystal S. Buprenorphine After Nonfatal Opioid Overdose: Reduced Mortality Risk in Medicare Disability Beneficiaries. *American Journal of Preventive Medicine*. 2023;65(1):19–29. doi:10.1016/j.amepre.2023.01.037

S204. Schoenfeld ER, Leibowitz GS, Wang Y, et al. Geographic, Temporal, and Sociodemographic Differences in Opioid Poisoning. *American Journal Of Preventive Medicine*. 2019;57(2):153–164. doi:10.1016/j.amepre.2019.03.020

S205. Siglin J, Sorkin JD, Namiranian K. Incidence of Postoperative Opioid Overdose and New Diagnosis of Opioid Use Disorder Among US Veterans. *American Journal on Addictions*. 2020;29(4):295–304. doi:10.1111/ajad.13022

S206. Smith AM, Acharya M, Hudson T, et al. Evaluating the temporal association between the recency of prescribed controlled substance acquisition and fatal and non-fatal opioid overdose. *Journal of the American Pharmacists Association*. 2023;63(2):648–654.e3. doi:10.1016/j.japh.2022.12.023

S207. Smith AM, Peng C, Porter A, Martin BC. The role of prescribed controlled substance acquisition as potential triggers of opioid overdose: A case-crossover study. *International Journal of Drug Policy*. 2024;134doi:10.1016/j.drugpo.2024.104620

S208. Tipping AD, Nowels M, Moore C, et al. Association of medications for opioid use disorder with reduced risk of repeat opioid overdose in Medicaid: A cohort study. *Journal of Substance Use and Addiction Treatment*. 2024;157doi:10.1016/j.josat.2023.209218

S209. Treitler P, Crystal S, Cantor J, et al. Emergency Department Peer Support Program and Patient Outcomes after Opioid Overdose. *JAMA Network Open*. 2024:E243614. doi:10.1001/jamanetworkopen.2024.3614

S210. Ward R, Obeid JS, Jennings L, et al. Enhanced phenotypes for identifying opioid overdose in emergency department visit electronic health record data. *JAMIA Open*. 2023;6(3)doi:10.1093/jamiaopen/ooad081

S211. Wei YJJ, Chen C, Fillingim R, Schmidt SO, Winterstein AG. Trends in prescription opioid use and dose trajectories before opioid use disorder or overdose in US adults from 2006 to 2016: A cross-sectional study. *PLoS Medicine*. 2019;16(11)doi:10.1371/journal.pmed.1002941

S212. Wei YJJ, Chen C, Schmidt SO, LoCiganic WH, Winterstein AG. Trends in prior receipt of prescription opioid or adjuvant analgesics among patients with incident opioid use disorder or opioid-related overdose from 2006 to 2016. *Drug and Alcohol Dependence*. 2019;204doi:10.1016/j.drugalcdep.2019.107600

S213. Yang H, Pasalic E, Rock P, Davis JW, Nechuta S, Zhang Y. Interrupted time series analysis to evaluate the performance of drug overdose morbidity indicators shows discontinuities across the ICD-9-CM to ICD-10-CM transition. *Injury Prevention*. 2021;27(S1):i35–i41. doi:10.1136/injuryprev-2019-043522

S214. Yunusa I, Gagne JJ, Yoshida K, Bykov K. Risk of Opioid Overdose Associated with Concomitant Use of Oxycodone and Selective Serotonin Reuptake Inhibitors. *JAMA Network Open*. 2022;5(2):E220194. doi:10.1001/jamanetworkopen.2022.0194
